# *Fusarium oxysporum* assisted green synthesis of small-sized silver nanoparticles for high antibacterial, and photocatalytic decolorization performances

**DOI:** 10.1186/s12866-024-03686-7

**Published:** 2025-01-06

**Authors:** Reyad M. El-Sharkawy, Inas A. Ahmed, Taghrid G. Kharboush

**Affiliations:** 1 Botany and Microbiology Department, Faculty of Science, Benha University, Benha, Egypt; 2 Department of Medical Biochemistry and Molecular Biology, Faculty of Medicine, Benha University, Benha, Egypt; 3 Central Laboratory for Research, Faculty of Medicine, Benha University, Benha, Egypt; 4 Department of Medical Microbiology and Immunology, Faculty of Medicine, Benha University, Benha, Egypt

**Keywords:** *F. oxysporum*, Silver nanoparticles, Multidrug-resistant, Anticancer, Antioxidant, Photocatalysis, *E. faecalis*, *P. aeruginosa*, *E. coli*

## Abstract

**Background:**

Novel platforms using nanotechnology-based medicines have exponentially increased in our daily lives. The unique characteristics of metal oxide and noble metals nanoparticles make them suitable for different fields including antimicrobial agents, cosmetics, textiles, wound dressings, and anticancer drug carriers.

**Methods:**

This study focuses on the biosynthesis of small-sized SNPs using exo-metabolites of *Fusarium oxysporum* via bioprocess optimization using Plackett-Burman (PBD) and central composite designs (CCD) while evaluating their multifaceted bioactivities.

**Results:**

The successful biofabrication of smaller-sized SNPs with an average particle size of ~ 5 nm was achieved upon the bioprocess optimization. The developed SNPs exhibited significant antibacterial activity against multidrug-resistant bacterial pathogens in a concentration- and time-dependent manner. The minimum inhibitory concentrations (MICs) for SNPs were 0.078 µg/ml (*Escherichia coli*), 0.156 µg/ml (*Pseudomonas aeruginosa*), and 1.25 µg/ml (*Enterococcus faecalis*), while the minimum bactericidal concentrations (MBCs) were correspondingly 0.156 µg/ml, 0.312 µg/l, and 1.25 µg/ml. SNPs-treated cells displayed bacteriostatic and bactericidal effects as revealed by time-kill assay and the ultrastructure changes observed in SEM and TEM analyses. The results marked the potent antioxidant activity of SNPs against DPPH, O_2_^•−^, H_2_O_2_, and OH-radicals with IC_50_ values of 74.3, 96.7, 116.6, and 167.9 µg/ml, respectively. Significantly, the biosynthesized SNPs displayed cytotoxic activity on MCF-7, A549, and HepG-2 cell lines with IC_50_ values of 89.4, 121.4, and 138.9 µg/ml, respectively. SNPs exhibited promising photocatalytic efficiency at different concentrations and times compared with dark conditions. The highest decolorization percentage of crystal violet dye was 98.60% after 240 min at 100 µg SNPs concentration.

**Conclusions:**

The green synthesis of SNPs by *F. oxysporum* exometabolites is eco-friendly, and inexpensive, with the production of small-size, and greatly stabilized nanoparticles. This study corroborated that SNPs can be highly promising enough to be applied for antibacterial and anticancer control systems, for ameliorating free radical-related disorders, and as a photocatalyst for wastewater treatment.

**Supplementary Information:**

The online version contains supplementary material available at 10.1186/s12866-024-03686-7.

## Introduction

Unresponsiveness of pathogenic microbes toward various antimicrobial drugs has become a major health concern of the twenty-first century, regarding a prime successful diagnosis and treatment of infectious microbes [1–4]. The current armory (antibiotics and other antimicrobials) may lose their effectiveness within 5 years. More than 70% of microbial diseases are caused by multi-drug-resistant pathogens, which show in vitro resistance toward one or more drugs in more than two antimicrobial categories [3, 5–7]. This may be attributed to the rapid genetic changes of the bacteria, reduction in membrane permeability, inactivation or enzymatic degradation of drugs, and modification of target proteins in pathogens which are responsible for infectious diseases. The efflux pumps may contribute to the extrusion of various antimicrobial agents among multidrug-resistant (MDR) pathogens [4, 8–11]. Hence, the production of non-conventional, safe, and efficient antimicrobial drugs based on nanotechnology has exponentially emerged as a new platform to overcome MDR [8, 12, 13].

Numerous physical and chemical processes have been employed for the generation of nanoparticles; however, those methods involve high cost and generate toxic chemicals and wastes that are not eco-friendly [14–17]. Currently, the generation of silver nanoparticles is mainly realized on the biological synthesis approaches using fungal, bacterial, and plant extracts, since the biosynthesis methods are facile, fast, environmentally friendly, cost-effective, and nontoxic. Moreover, the addition of capping agents is not needed for the biological method which in turn simplifies the generation process [15, 18–21].

To date, fungi are the most attractive hotspot to discover novel medication candidates, antimicrobials, and metabolites as they are characterized by their efficiency, sustainability, and ability to produce various biomaterials when compared to other biosystems [22–24]. The generation of SNPs by harnessing exometabolites of fungi is globally preferred over other bioagents (e.g. bacteria) due to their high growth rate, high tolerance to heavy metals, production of great amounts of proteins, mass production of nanoparticles, little poisonousness of residues, ease handling and low cost of down streaming [19, 24]. The application of fungal enzymes instead of chemical processes in the biosynthesis process facilitated making the process eco-friendly via the reduction of the negative influence on the environment.

Various studies have reported the successful biosynthesis of SNPs using the fungal water extract of *Rhizopus stolonifer* [25], *Fusarium oxysporum* [27], *Aspergillus niger* [28], and *Penicillium oxalicum* [29]. Among these fungi, Fusaria species are the prime selection for scientists as evident from the available literature. Fusaria species play a vital role in the green synthesis of SNPs and can be deemed nanofactory for the SNPs synthesis. The high potential for the biofabrication of SNPs was reported for *F. oxysporum* among various Fusaria species [23]. The reduction of aqueous Ag^+^ into SNPs is performed by NADH-dependent nitrate reductase enzyme which is secreted by *F. oxysporum*. The surface charge, shape, size, and the used metal concentrations are the main factors determining the toxicity of nanomaterials, hence, the green synthesized SNPs using exometabolites of Fusaria species can be widely employed in different fields, particularly agriculture and medicine.

The release of toxic recalcitrant dyes into the surrounding environment via different industrial effluents is one of the major environmental problems in developing countries [15]. The photocatalytic activity of nanocatalysts is mainly correlated to their specific area [15, 17]. The nanocatalysts with higher surface area and smaller sizes can enhance the adsorption and catalytic efficiency of nanomaterials. Hence, the reduction in the nanoparticle sizes’ is of promising interest owing to the highly degrading ability of dyes [6, 15].

The current study aimed to synthesize small-sized silver nanoparticles using an eco-friendly approach through harnessing the cell-free exometabolites of *F. oxysporum* as a biocatalyst. The mode of action against bacteria at a cellular level, and the antioxidant, and anticancer activities of SNPs were investigated.

## Materials and methods

### Bacterial cultures, and chemicals

Bacteria were isolated from urine samples, collected from the Urology Department, Faculty of Medicine, Benha University, Egypt. Patients taking antibiotic treatment within the previous 3 days were excluded from the current work. Under aseptic conditions, the collected samples were transported into the Microbiology Lab (Faculty of Medicine, Benha University, Egypt). The developed bacterial cultures were grown till reaching the mid-log phase and then the concentration was adjusted to 10^6^ colony-forming units (cfu)/ml. All chemicals were obtained from Sigma-Aldrich and were of analytical grade.

### Isolation of fungal strains and biosynthesis of silver nanoparticles

Different fungal isolates used in the present study were isolated from various soil samples collected from Qalyubia governorate (Benha, Egypt) on Czapek’s-Dox agar (CDA) medium. The developed fungal isolates were picked up, purified through re-inoculation onto CDA medium, and subsequently stored onto the same medium for further study [16, 19]. The potentiality of such fungal isolates for their biosynthetic capability of SNPs was examined using a modified medium containing (g/l): 0.6 yeast extract; 10 glucose; 2 K_2_HPO_4_; 0.1 MgSO_4_.7H_2_O; 1 (NH_4_)_2_SO_4_. After incubation at 28 °C for 5 days and under sterile conditions, the mycelium was harvested by filtration from a medium and washed thoroughly using sterile distilled water. The fungal mycelium (5.0 g) was added into the 50 ml deionized water, mixed well, and incubated in the previous conditions. Mycelium was separated by filtration, and centrifuged. The free-cell filtrate was then amended with 1.0 mM of AgNO_3_. After incubation at 28 °C and 100 rpm for 5 days in dark conditions, the biosynthetic SNPs were collected throughout centrifugation at 8,000 rpm for 10 min. To recover the green synthesized SNPs, repeated centrifugation cycles were performed for 20 min at 8,000 rpm, followed by washing in deionized water, and ultimately the collected nanoparticles were dried. In parallel, negative control (silver nitrate alone) and blank (fungal supernatant without the addition of AgNO_3_) were performed in the same conditions [21, 30]. The SNPs-biosynthetic process was monitored via the visual inspection of the color change from yellow to dark reddish-brown color of the mixture preparation [30]. The concentration of silver in the resulting SNPs was calculated according to [24].

### Molecular identification of the potent fungal isolate synthesizing SNPs

The fungal isolates were examined for morphological, cultural, and microscopical features and then identified according to the standard keys [31–33]. The molecular identification of the promising SNPs producer was performed according to the entire sequence of the internal transcribed sequence (ITS) of the rDNA region [16, 19]. The genomic DNA was extracted and amplified using primers of ITS1 (5’-TCC GTA GGT GAA CCT GCG G-3’) and ITS4 (5’-TCC TCC GCT TAT TGA TAT GC-3’) [34]. The PCR mixture (50 µl) contains 2 µl extracted genomic DNA, 1 µl of each primer, 2.5 U Taq DNA, 0.5 mM MgCl_2_, and 2× PCR master mixture (AlphaDNA Co, Canada). The PCR was accomplished in a Solgent EF-Taq, PCR Machine name: 9700(ABI), MJ research thermal cycler (USA) with a hot starting conducted at 95 °C for 3 min, followed by 35 cycles of 95 °C for 30 s, annealing at 50 °C for 30 s and extension at 72 °C for 90 s and then a final extension 72 °C for 5 min. The PCR amplicons were checked using 1% agarose gel and then sequenced by the same primer sets. To reveal the phylogenetic position of the fungal isolate, the obtained sequence was compared with the ITS sequences in the GenBank database using the BLAST tool. Multiple sequence alignment was conducted by importing the sequence into MEGA-X 11 and a phylogenetic tree was constructed with a confidential level of 1000 bootstrap using the neighbor-joining method.

### Process factors optimization for small-sized SNPs production

To determine the significant variables affecting the production of SNPs using the promising fungal water extract, the influence of six independent variables, namely pH, temperature, incubation time, silver precursor concentration, biomass amount, and the ratio of fungal extract to and silver nitrate, on the biosynthetic process was assessed by the 2-factorial Plackett-Burman design (PBD) [3, 8, 13, 16, 29]. The level of each parameter is illustrated in Table 1. Significant four independent variables influencing the biosynthetic process of SNPs were optimized by CCD of Response Surface Methodology (RSM) to investigate the individual and mutual interaction of the tested factors [5, 16] (Supplementary materials). The statistical significance of the tested factors was explored by appraising the *F*-value model, the confidence level, and the *P*-value [15]. Variables with *P* < 0.05 at 95% level displayed a significant behavior and were involved in the analysis.

**Table 1:** Codes, independent variables and their two levels employed for the SNPs production by harnessing the exo-metabolites of *F. oxysporum* using Plackett-Burman design

Code	Synthesis variable	Level	
		-1	+ 1
X_1_	pH	5	10
X_2_	Temperature (°C)	20 °C	50 °C
X_3_	Time (min)	0	60
X_4_	Ag precursor concentration (mM)	0.5	2
X_5_	Biomass amount (g)	5	10
X_6_	Ratio of AgNO_3_: Fungal filtrate	1:1	1:2

### Characterization of the biosynthetic silver nanoparticles

The process of silver ion bio-reduction was investigated spectroscopically at the wavelength range 300–800 nm using a UV-vis spectrophotometer, normalized to the untreated fungal filtrate and AgNO_3_. The as-formed SNPs were placed in a 1.0 cm quartz cuvette (path length), and the equipment resolution was 1.0 nm. The preparation was scanned in a wavelength of 300 to 800 nm. The crystallinity of the biosynthetic SNPs was affirmed using X-ray powder diffraction (XRD, X’PERT PRO, MiniFlex 300/600 X-ray, USA) which operated with Cu-Ka radiation source at 28 °C and 40 kV. The preparation was scanned at a 10.00° min scanning rate in the range of 20–80° of 2θ. The size and morphology of the obtained particles were investigated using a transmission electron microscope (TEM). TEM imaging analysis was carried out using a JEOL JEM-1010 TEM operated at 100 kV. In brief, the prepared nanoparticle drops (5 µl) were diluted using distilled water, subsequently dropped, covered upon freshly carbon-coated copper grids (400 mesh), evaporated, and dried at ambient temperature. The elemental analysis of the green SNPs was determined using a scanning electron microscope (JEOL JEM-1010 SEM) attached to an energy-dispersive X-ray detector (SEM-EDX) at 10 keV. Using carbon tape, powdered samples were adhered to the aluminum stub for analysis via the secondary electron technique. The incorporation of different functional groups present in the fungal extract was monitored via Fourier-transform infrared (FTIR). Samples (30 µl) were placed on an FTIR spectrometer. Transmittance measurements were performed in the range from 500 to 4000 cm^− 1^ at 4 cm^− 1^ resolution.

### Antibiotic susceptibility test

The antibiotic susceptibility testing was performed by the disc diffusion method [35]. Twelve antibiotic discs (Oxoid, UK) namely cefotaxime (CTX, 30 µg), Azthromycin (AZM, 30 µg), penicillin (G, 10 µg), ampicillin-sulbactam (SAM), cefepime (FEP, 30 µg), amoxicillin-clavulanic acid (AMC, 20 + 10 µg), cefuroxime (CXM), Imipenem (IPM, 10 µg), Cefoxitin (FOX, 30 µg), amikacin (AK, 30 µg), levofloxacin (LEV, 5 µg), and ciprofloxacin (CIP, 30 µg), were chosen for assaying the antimicrobial susceptibility of the collected sample. The inhibition diameter is inferred based on the Clinical and Laboratory Standards Institute (CLSI) guidelines (2020) [36].

### Molecular characterization of the multidrug-resistant bacteria

A selected set of multidrug-resistant bacteria, i.e. EG-MDR#3, EG-MDR#11, and EG-MDR#18 was identified based on their 16 S-rRNA gene sequencing. The bacterial genomic DNA was extracted and used as a template for amplification by performing colony PCR with the primers of 27 F (5′-AGA GTT TGA TCC TGG CTC AG-3′) and 1492R (5-CGG TTA CCT TGT TAC GAC TT -3′) [37, 38]. The PCR was accomplished using Solgent EF-Taq, PCR Machine name: 9700(ABI), MJ research thermal cycler (USA) in 50 µl PCR reaction. The PCR products were purified and sequenced using the forward and reverse primers, followed by sequencing, and the obtained sequences were compared with the NCBI database using the BLAST tool. For phylogenetic analyses, the 16 S-rRNA sequences-based phylogenetic tree was conducted with the neighbor-joining method. The confidential level was 1000 bootstrap replicates [39].

### Assessment of the antibacterial activity of SNPs

The antibacterial activity of SNPs was evaluated by the Kirby-Bauer disc diffusion method according to [40]. In brief, 0.1 ml of the overnight bacterial culture (10^6^ cfu/ml) was spread onto Muller-Hinton agar (MHA, Merk, Germany). Sterile discs (8 mm) were ultrasonically coated for 30 min with 30 µl of various SNPs solution (0.078, 0.156, 0.312, 0.625, 1.25, 2.5, 5, 10 µg/ml), and then were placed on the solidified surface of MHA plate. Subsequently, the plates were incubated for 28 h at 37 °C. The zones of inhibitions were measured and then interpreted according to the Clinical and Laboratory Standards Institute (CLSI) guidelines (2020) [36].

### Determination of MIC and MBC

The MIC determination was carried out based on the method of CLSI (2020) with some modifications. The assay was performed in a 96-well microtiter plate (Sigma Aldrich, USA) using inocula concentrations of 10^6^ (cfu/ml). SNPs (10 µg/ml), which were prepared in 1.0% DMSO, were mixed with the investigated bacteria in a 2-fold dilution using MH broth. The well in columns 1.0 and 2.0 containing, respectively, MH broth and MH broth and SNPs were defined as negative controls, while the well in column 3 containing medium and the respective bacteria was demanded as positive growth control. The solution of resazurin (30 µl) was then added to each well of the plate and incubated overnight at 37 °C. The growth of bacteria was determined by the color change from purple/blue to colorless/pink. The MIC was described as the lowest concentration of the SNPs which inhibited the visible growth [8], whilst the minimum bactericidal concentration was defined as the lowest concentration which killed the bacterial culture [10, 20]. The MBC determination was conducted by subculturing (10 µl) of the two lowest concentrations in microtiter plates, showing no visible growth in the MIC test, on MHA plate for 24 h at 37 °C.

### Kill-Time curve assay

The time-kill analysis was determined according to the CLSI guidelines using MH broth [41]. Before the experiment, bacterial isolates were inoculated into MH broth to obtain a bacterial suspension with a concentration of 10^6^ cfu/ml, as ascertained by viable counts. Each bacterial species was tested against the 0 × MIC, ½ × MIC, 1 × MIC, and 2 × MIC values, obtained from mixing the above solution of MH broth and bacteria with the SNPs solution in a final volume (one ml). The MH broth was incubated aerobically for 24 h at 37 °C. At selected time intervals (0, 0.5, 1, 2, 3, and 4 h), aliquots (100 µl) of suspension each were taken and plated onto MHA. The viability of the tested isolates was determined in terms of cfu/ml.

### Morphological characterization of *E. coli* treated with SNPs

For studying the morphological changes on exposure to SNPs, the most susceptible bacterial strain was treated with SNPs at the MBC dose. After incubation at 37 °C for 24 h, the cells were pooled by centrifugation at 5,000 rpm for 10 min. The pellets were washed with sterile distilled water. For bacterial cell visualization using SEM, the pellets were fixed at 4 °C for 24 h with 2.5% glutaraldehyde-phosphate buffer at pH 7.0). The preparation was subsequently serially dehydrated using different ethanol concentrations deposited on a coverslip and for 24 h underwent vacuum drying. The analysis was performed by SEM (JEOL- JSM-6510LV microscope Japan).

For ultrastructure analysis, the overnight bacterial culture was incubated with SNPs at the MBC concentration for 24 h at 37 °C. The preparation was centrifuged for 10 min at 5,000 rpm and then washed thrice with buffer solution. The cells were primarily fixed in 2.5% glutaraldehyde-phosphate buffer at pH 7.0 for 1 h at 4 °C, while at the last stage were post-fixed with osmium tetra-oxide (1.0%). The sample was then washed several times with phosphate buffer, and subsequently dehydrated in 15-minute intervals using different ethanol concentrations (30–100%), finally the dehydrated cells were dried using a desiccator, and coated with gold before examination in TEM (JEOL- JEM-1010LV microscope Japan). In parallel, the bacterial strain (without SNPs) was processed under the same conditions and considered as a control.

### Determination of antioxidant activity of SNPs

#### DPPH radical scavenging activity

The antioxidant activity of the biosynthesized SNPs and a reference standard (ascorbic acid) was measured by DPPH (2,2-diphenyl-1-picrylhydrazyl) free radical assay [42]. In brief, different concentrations of SNPs and ascorbic acid (25–250 µg/ml) were individually prepared and mixed with ethanolic DPPH (0.1 mM). The prepared mixture was vigorously agitated and then incubated for 20 min in the dark. For each test tube, the absorbance was monitored at 517 nm using a UV-visible spectrophotometer. A control sample containing an ethanolic solution of DPPH without SNPs was conducted under the same conditions, while a blank of ethanol alone was used. The antioxidant activity was expressed in terms of % DPPH scavenging inhibition which was determined from the following equation:

1 $$\:\text{I}\text{n}\text{h}\text{i}\text{b}\text{i}\text{t}\text{i}\text{o}\text{n}\:\left(\text{\%}\right)=\:\frac{{A}_{C}-\:{A}_{S}}{\:{A}_{C}}\:\:\times\:100$$

Where, $\:{A}_{C}$ is the absorbance of control and $\:{A}_{S}$ is the absorbance of sample.

#### Superoxide anion radical scavenging activity

The superoxide anion radical-scavenging pattern of SNPs was detectable using the method of [43] with slight modification. In brief, a reaction preparation contained 100 µM nitroblue tetrazolium (NBT), 100 mM phosphate buffer pH 4.7, 5 µM riboflavin, and 15 mM methionine, was mixed with different concentrations of SNPs (25–250 µg/ml). The preparations were incubated at ambient temperature for 15 min in front of a fluorescent lamp. The absorbance was monitored at 560 nm. The scavenging capability was determined using the equation mentioned for the DPPH assay.

#### Hydrogen peroxide scavenging activity

The potentiality of SNPs to scavenge hydrogen peroxide (H_2_O_2_) was investigated based on the method of [44] with some modifications. Briefly, the reaction mixture including 0.6 ml H_2_O_2_ (40 mM), 2.4 ml of phosphate buffer (100 M, pH 7.4), and varying concentrations of SNPs was prepared, agitated vigorously, and incubated for 10 min at Lab temperature. The absorbance was determined spectrophotometrically at 320 nm against phosphate buffer without H_2_O_2_ (blank) and the concentration of H_2_O_2_ scavenging was detectable by the equation mentioned for the DPPH assay.

#### Hydroxyl radical scavenging activity

The ability of SNPs to scavenge hydroxyl radical was performed according to the method of [45] with some modification. Concisely, 0.2 ml of 1.0 mM FeCl_3_, 1.0 mM EDTA, 50 mM deoxy-D-ribose in potassium phosphate buffer (pH 7.4), 1.0 mM ascorbic acid, and 0.1 ml of varying concentrations of SNPs, subsequently 20 mM H_2_O_2_, vigorously shake, and incubated for 30 min at 50 °C. Further, 1.0 ml of trichloroacetic acid (2.8%, v/v) and 1.0 ml of 2-thiobarbituric acid (1.0%, w/v) were added to the preparation, shaken well, and heated for 30 min in a water bath at the previous temperature. A control that contains the preparation without SNPs was performed. The absorbance was determined at 532 nm against phosphate buffer and the hydroxyl radical scavenging was calculated with the formula used for DPPH assay.

#### Cytotoxic activity of SNPs

The cytotoxicity of the green SNPs versus MCF-7 cells were examined using human cells of hepatocellular carcinoma (HepG2), breast cancer (MCF-7) and lung carcinoma (A549) based on the 3-[4,5-dimethylthiazol-2-yl]-2,5-diphenyltetrazolium bromide (MTT) reduction assay analysis [17, 46]. Cells at a density of 1 × 10^4^ cells per well were cultured in a sterile 96-microtitre plate. After overnight incubation at 37 °C, different concentrations (100 µl, 0–250 µg/ml) of biosynthesized SNPs were supplemented to cultured media. The preparations were incubated at 37 °C for 24 h, and subsequently 10 µl of MTT solution (5 mg/ml PBS) was performed to each well and further incubated under a humidified atmosphere with 5% CO_2_ at 37 °C for 3 h. The media and MTT solution were then aspirated, and the formed formazan was suspended in 100 µl of dimethyl sulfoxide (DMSO). After 15 min, the absorbance was determined at 570 nm using a microplate reader (680 XR reader, Bio-Rad, Hercules, CA, USA). In parallel, the media only served as negative control, while the cells cultured without sample served as positive control. The experiments were performed in triplicate. The growth inhibition (%) was calculated using the following equation:

2 $$\text { Cell viability }(\%)=\frac{\mathrm{OD}_{\mathrm{c}}-\mathrm{OD}_{\mathrm{s}}}{O \mathrm{D}_{\mathrm{c}}} \times 100$$

where$\:{\text{O}\text{D}}_{\text{C}}\:$is the optical density of the control, and$\:{\text{O}\text{D}}_{\text{S}}\:$ is the optical density of sample.

#### Crystal violet-photocatalytic degradation using SNPs

The photocatalytic efficiency of SNPs for degrading crystal violet (CrV) dye as a model at various stimulation conditions (dark and sunlight irradiation) was investigated in batch experiments according to [47]. In brief, the photocatalytic experiment was carried out during summer under sunlight irradiation (during the sunny and bright days) at the daytime from 10 am to 2 pm in open air. The decolorization experiments were performed in 250 ml Erlenmeyer flasks containing 50 ml of CrV aqueous solution with a concentration of 100 mg/l, mixed with SNPs concentration of 50 µg and 100 µg at various contact times (30, 60, 120, and 240 min). Before the photocatalytic investigation, the preparations were incubated in the dark for 30 min at ambient temperature with a continuous swirling to attain absorption/desorption equilibrium among the photocatalyst and CrV dye. For relative investigation, another mixture was performed in dark. The decolorization percentages was determined by withdrawing 1.0 ml of the preparation, centrifuged for 3.0 min at 8000 ×g and the optical intensity was measured using spectrophotometer at λ_max_ 558 nm. The CrV removal percentages were calculated by the following formula:

3 $$\mathrm{R} \%=\frac{\mathrm{CrV}_i-\mathrm{CrV}_f}{\mathrm{CrV}_i} \times 100$$

where R% is the crystal violet removal (%), $\:{CrV}_{i}$ is the initial CrV absorbance, $\:{CrV}_{f}$ is the final CrV absorbance.

### Data processing analysis

The experiments were performed in triplicate unless otherwise mentioned and the obtained data were represented as means ± standard deviation (SD). The number of colonies was represented as Log_10_ cfu/ml. The SNPs optimization process was carried out by applying PBD and CCD on the investigated independent factors via MINITAB 21.0 statistical software package, USA.

## Results and discussion

### Biosynthesis of silver nanoparticles (SNPs)

Among twenty-two fungal isolates obtained from the collected soil samples, the cell-free filtrate of nine fungal isolates was capable of the green synthesis of the silver nanoparticles. These fungal isolates were identified according to the universal keys. The most promising fungal isolate showing the highest SNPs yield was selected for further work and nominated as EG-AR3. The process of silver ions reduction using the cell-free filtrates was initially investigated through the color change to a reddish brown color, compared to the control, revealing the Ag^+^ reduction by various biomolecules including proteins, enzymes, polysaccharides, vitamins, and amino acids [2, 11, 15, 48].

Fungi can produce various bioactive compounds that can be employed in various applications. They are characterized over other microorganisms, by their ability to tolerate, internalize, and bioaccumulate heavy metals. They can be broadly engaged as capping and stabilizing agents in different biological processes. They can largely produce enzymes and proteins that play an important role in the production of stable and small-sized nanomaterials [11, 23]. The biosynthesis of SNPs using microorganisms can be performed either by extracellular or intracellular methods [5, 39, 49, 50]. However, the extracellular synthesis of nanoparticles is preferable over the intracellular one due to its ease and rapid production, as well as facile purification. Extracellular biosynthesis was employed in the current research as a conventional and advantageous method. The extracellular production of the silver nanoparticles using various microorganisms was reported by [7, 41, 51, 52] who found the color shift from pale yellow to deep brown color when incubating 1.0 mM AgNO_3_ with the cell-free supernatant.

### Molecular identification

**Fig. 1:**
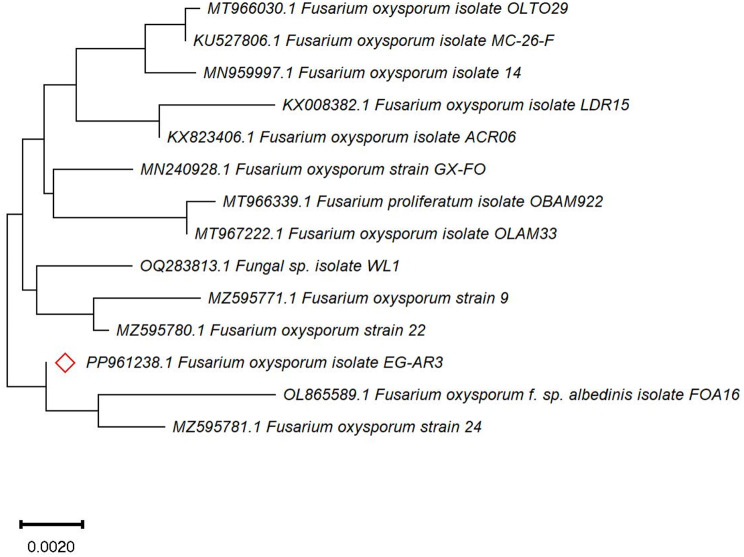
Phylogenetic analysis of *F. oxysporum* EG-AR3 sequence with close sequences from NCBI was performed using MEGA-X 11 via the Maximum Likelihood model and default settings. The symbol  refers to the isolate in the present study. The bar length denotes 0.002 substitutions for each nucleotide site

The morphological identity of *F. oxysporum* EG-AR3 was further affirmed according to the entire sequence of the internal transcribed sequence (ITS) of the rDNA region. The amplicon was nominated as *F. oxysporum* based on the BLAST searching tool of the NCBI database (Fig. 1). The sequence was deposited in the database with accession number PP961238.1 with a similarity percentage of 99.0% with the isolates of *F. oxysporum* which had accession numbers OL865589.1, MZ595781.1, MZ595780.1, and OQ283813.1 with zero E-value and 99% query coverage.

### Biosynthesis of smaller-sized SNPs via bioprocess optimization

**Fig. 2:**
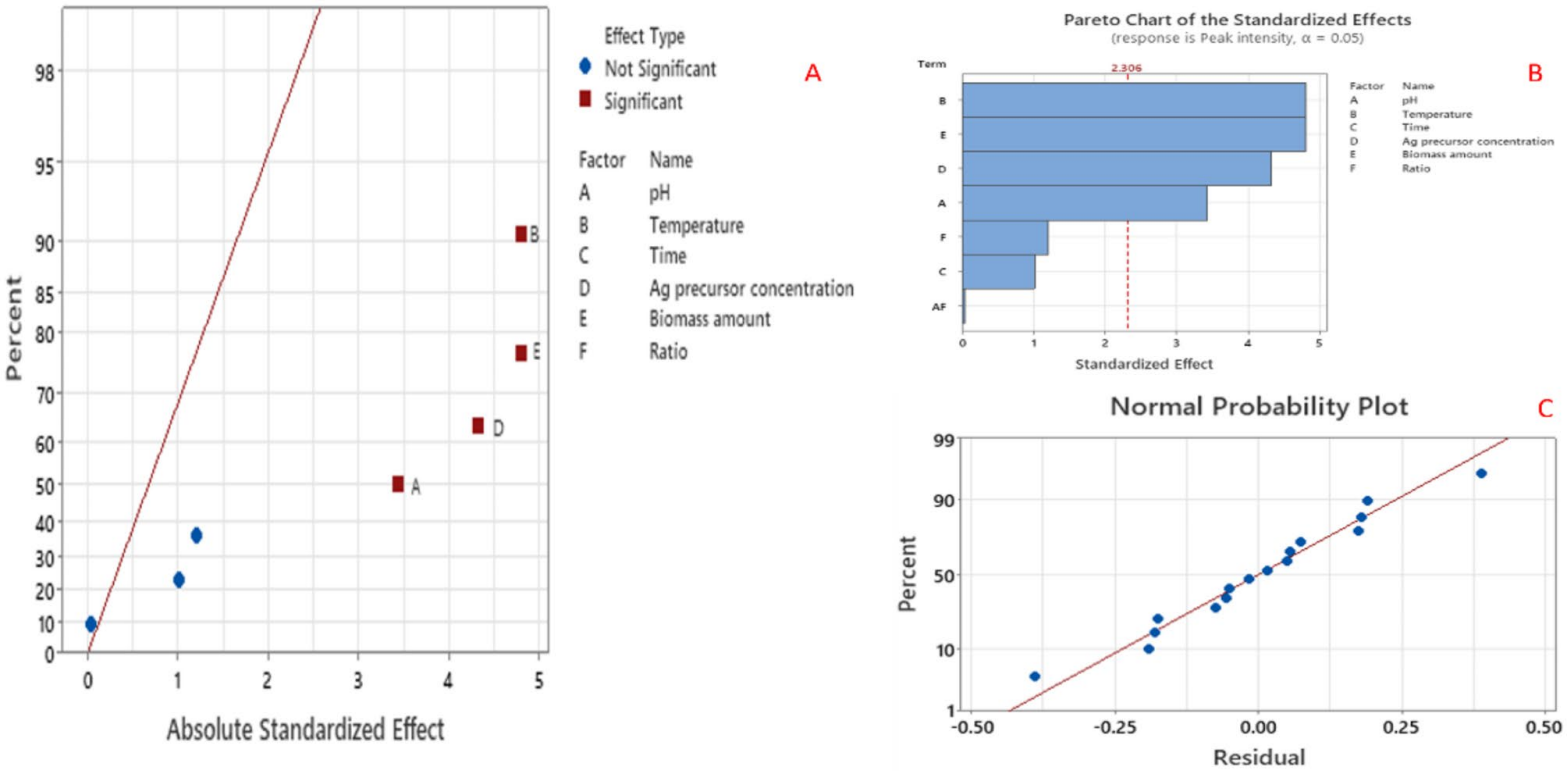
The impact of various input variables on the green production of SNPs using *F. oxysporum* based on the design of Plackett-Burman. (**A**) Standardized effect plot of the examined variables for SNPs synthesis, (**B**) Plot of Pareto chart displayed the sequence of significant of individual factor, (**C**) Plot of the normal probability of the input factor based on the first order equation

Plackett-Burman screening of six independent sixteen-trial designs was performed to determine the significance of the tested factors on the biosynthetic process of SNPs using the biomolecules harnessing from the *F. oxysporum* filtrate [26, 30, 53]. The predicted and consistent experimental values along with the significance of the input parameters on the SNPs production are illustrated in Table S1. The variation among the actual and predicted values of PB design showed the importance of process optimization on the SNPs production by the water extract of *F. oxysporum*. The maximum SNPs production has been detected at the trial # 4 with the incorporation of pH (5, -1), temperature (20 °C, -1), time (60 min, + 1), Ag precursor concentration (2 mM, + 1), biomass (5, -1), and the ration of AgNO_3_ to the fungal extract (1:1, -1), however, the minimum SNPs production have been determined at the trial number # 12 with the variables of pH (10, + 1), temperature (50 °C, + 1), time (60 min, + 1), Ag precursor concentration (2 mM, + 1), biomass (10 g, + 1), and the ration of AgNO_3_ to the fungal extract (1:2, + 1). Analysis of variance (ANOVA) and multiple regression statistics of the input variables on the SNPs production process were evaluated. Temperature displayed the highest effect on the SNPs synthesis process as revealed from Fig. 2A. The significance of each factor of the input variables on the SNPs bioproduction process was clearly illustrated in the Pareto Chart (Fig. 2B). The organization of the residual points adjacent to the diagonal line, reveals the normal distribution of independent variables and the accurate fitting of the actual and predicted values (Fig. 2C). According to ANOVA analysis of PB design, the model was greatly significant as revealed from the probability *P*-value of 0.001 and the Fishers *F*-teste of 11.31. After neglecting the non-significant variables (*P* > 0.05), the regression equation for SNPs synthesized by using *F. oxysporum* biomolecules was:

4 $$\eqalign{& {\rm{SNPs\,by\,F}}\,{\rm{. oxysporum \,(Peak\,intensity, nm) = 4}}.{\rm{9995 - 0}}.{\rm{0880pH - 0}}.{\rm{02050}} \cr & {\rm{ Temperature }} + {\rm{ 0}}{\rm{.00217\,Time }} - 0.3700\,{\rm{Ag\,precursor\,concentration - }}0.1230 \cr & {\rm{ Biomass\,amount }} - 0.070\,{\rm{ Ratio }} - 0.0010\,{\rm{p}}{{\rm{H}}^*}{\rm{ Ratio }} \cr}$$

### Optimization of SNPs synthesis using CCD

**Fig. 3:**
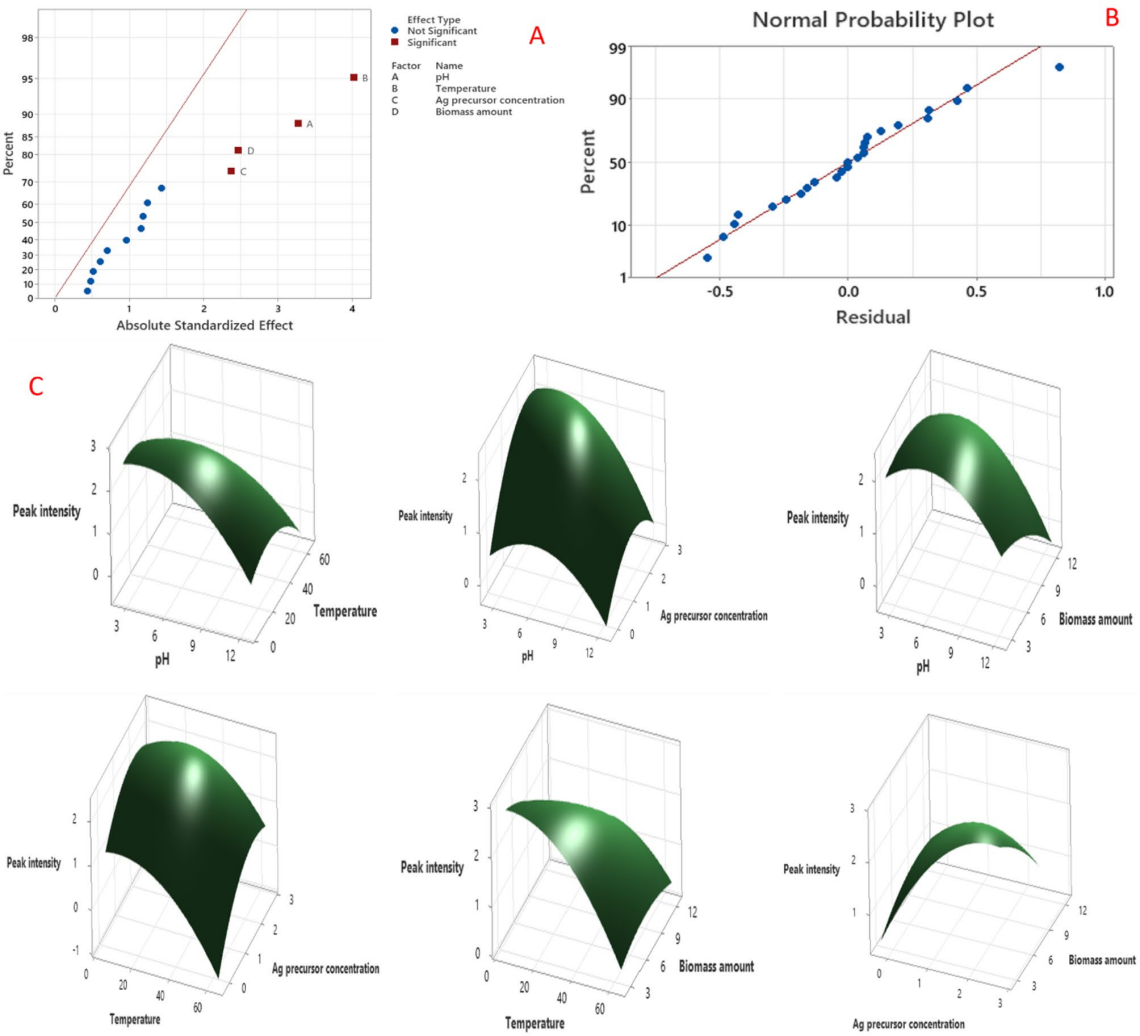
Plots of probability and 3D-response surface for the SNPs production using the water extract of *F. oxysporum* based on the design of CCD in RSM. (**A**) Plot of standardized effect of each variable on the SNPs biosynthetic process, (**B**) Plot of residuals vs. normal probability (%), (**C**) 3D-surface response illustrating the interaction effect of various input variables on the SNPs biosynthetic process using the water extract of *F. oxysporum*

The major factors affecting the biosynthesis of SNPs using the biomolecules of *F. oxysporum* were further optimized by using the design of RSM (CCD) [5, 10, 54]. Upon optimization of various independent variables using CCD design, the most significant parameters were pH (*p* = 0.009), temperature (*p* = 0.001), the concentration of Ag precursor (*p* = 0.003), and biomass amount (*p* = 0.001) (Fig. 3A). On the contrary, the non-significant factors were time, and the ratio of fungal extract to silver nitrate. According to the CCD results, the highest response (peak intensity) was determined in trial # 5 with the preparation at pH (5, -1), temperature (20 °C, -1), metal precursor (2 mM, + 1), and biomass amount (5 g, -1) (Table S2). The model is highly significant based on the *F*-value of 3.24 and *P*-value of 0.034 (*P* < 0.05). The residual points were organized around the diagonal line as illustrated in the plot of normal probability (Fig. 3B), hinting at the intact fitting of the predicted SNPs yield and the experimental values. Graphs of 3D-surface response were plotted based on the interaction of constant values to the three input factors of the design (Fig. 3C). According to ANOVA, the significant variables (*P* < 0.05) are pH, temperature, silver precursor, and biomass amount (Table 2), but not the variables’ interaction. The 3D graphs demonstrated that the SNPs biosynthetic process was remarkably enhanced in the central value of each tested variable. The developed model has a nonsignificant lack-of-fit value of 13.718, hinting that the model is acceptable. The high coefficient of determination (R^2^ = 0.9982) could justify a significant variance in the design space. From the obtained results, the proposed model can be expressed by the 2nd -order equation:

5 $$\eqalign{& {\rm{SNPs\,by\,F}}\,{\rm{.oxysporum\,(Peak\,intensity,nm) }} \cr & = 0.60 + 0.352{X_1} - 0.0225{X_2} + 1.92{X_4} + 0.135{X_{5 - }} \cr & - 0.0296X_1^2 - 0.000760{X_{22}} - 0.311X_4^2 \cr & - 0.0104X_5^2 + 0.00233X_1^*{X_2} - 0.0407X_1^*{X_4} \cr & - 0.0006X_1^*{X_5} + 0.0057X_2^*{X_4} \cr & + 0.00320X_2^*{X_5} - 0.0953X_4^*{X_5} \cr}$$

**Table 2:** ANOVA analysis of CCD design for the interaction pH, temperature, metal precursor concentration, and biomass amount variables on the biosynthesis of SNPs using *F. oxysporum*

Variable code	Sum of squares		F-value	*P*-value
X_1_	2.64		10.6	0.008
X_2_	3.99		16.1	0.002
X_4_	1.39		5.6	0.039
X_5_	1.50		6.1	0.034
X_1_^2^	0.39		1.6	0.240
X_2_^2^	0.33		1.3	0.275
X_4_^2^	0.35		1.4	0.265
X_5_^2^	0.04		0.2	0.671
X_1_ × _2_	0.13		0.49	0.498
X_1_ × _4_	0.09		0.38	0.554
X_1_ × _5_	0.06		0.23	0.640
X_2_ × _4_	0.07		0.26	0.619
X_2_ × _5_	0.23		0.93	0.357
X_4_ × _5_	0.51		2.06	0.181

The SNPs production using the biological route was characterized by its easy performance and cost-effectiveness, however, the synthesis of SNPs with promising properties including stability, mono-dispersity, and small sizes, requires the optimization of variables in the production process [6, 26, 55]. Among the statistical methods used for the optimization of different factors in nanoparticle production, the design of PB and RSM models provides satisfactory information on the importance of each variable and reduces the repetitive runs for the process [16, 30]. PBD has been commonly employed for optimizing different biological processes to reduce the need for more repetitive trials [16].

Concurrently with our results, various variables in the synthesis of SNPs including pH, temperature, production time, and culture biomass fluctuated according to the type of microbes to achieve the desired properties of SNPs. Temperature had the most effect on the biosynthetic process, followed by pH and concentration of silver precursor [21, 30, 55]. Similarly, temperature is the main factor in the current study influencing the process of SNPs biosynthesis. It performed a very important role during the reduction process of silver ions. The reaction is highly affected under higher temperatures since it can reduce or inhibit the effectiveness of different reducing agents within the fungal supernatant. A higher amount of fungal biomass may improve the biosynthesis of SNPs owing to the existence of various reducing and stabilizing agents in the fungal filtrate. The rise in the concentration of the silver precursor leads to changes in the morphology and behavior of silver nanoparticles. It is reported that the SNPs tend to aggregate under too high a concentration of metallic silver [11]. The synthesis process of silver nanoparticles achieves a plateau during the reaction progress due to the reduction in silver precursor concentration and the reducing agent’s containing filtrate [56]. The particle size of SNPs is highly affected by the fluctuations in the pH values. The particle sizes are reduced by increasing the pH value within a specific timeframe. Spherical, small-sized, and monodispersed SNPs were obtained in higher amounts at a given pH range; however, a further rise in the pH value increases the nucleation and aggregation of the SNPs [47, 56]. Several studies recorded the optimization of different factors in the synthetic process to achieve the desired SNPs properties [2, 15, 18].

### Physicochemical characterization of SNPs

**Fig. 4:**
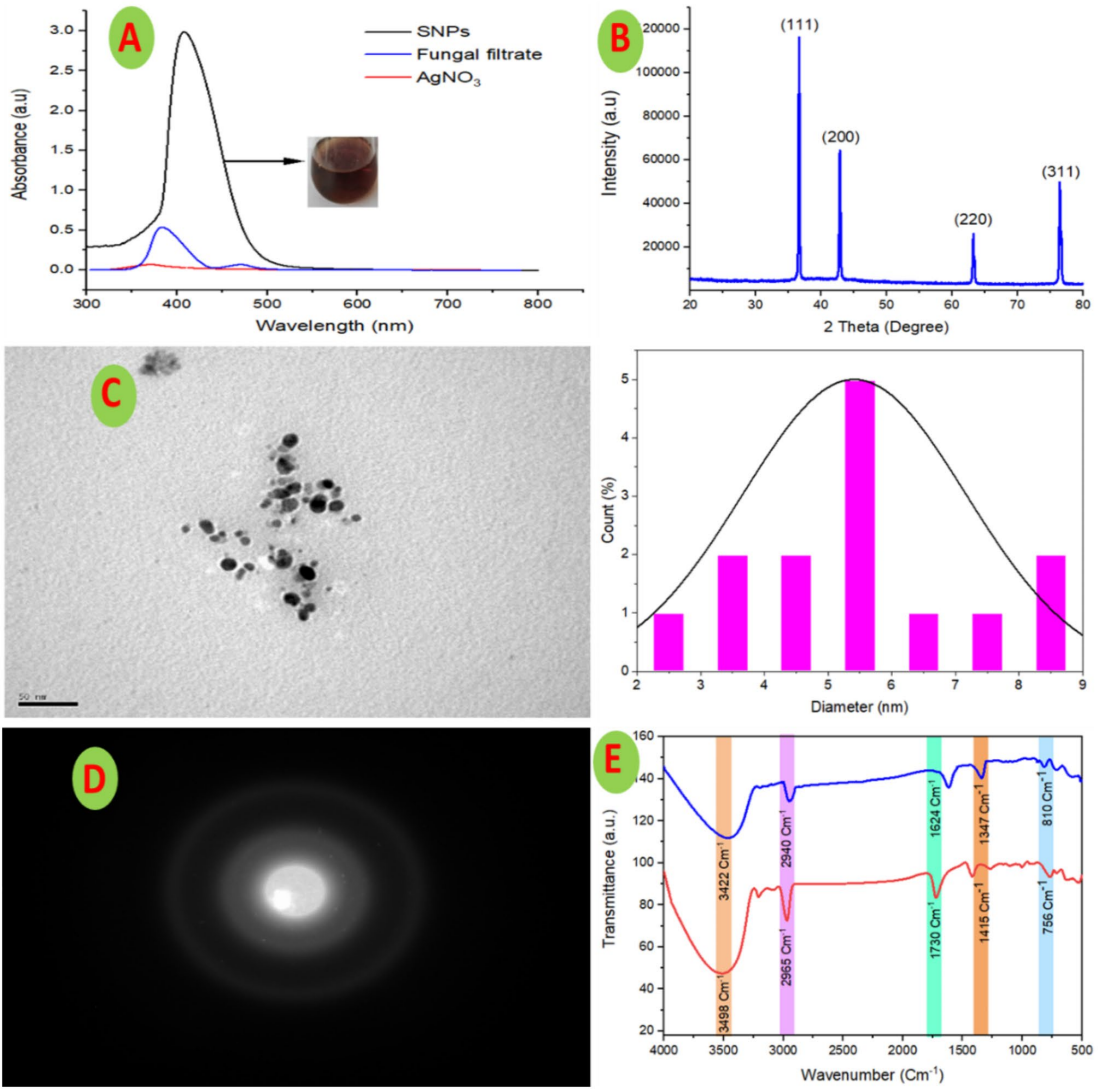
Uv-Visible spectroscopy (**A**), X-ray diffraction pattern (**B**), TEM images, scale bar = 50 nm (**C**), SAED (Selected area electron diffraction pattern) (**D**), FT-IR spectrum of fungal extract (red line) and biosynthesized SNPs (blue line) (**E**) of fungal extract and biosynthesized SNPs, obtained by green synthesis using *F. oxysporum*

Uv-visible spectra of the myco-synthesized SNPs (Fig. 4A) displayed a fluctuation in the surface plasmon resonances (SPR). While a characteristic SPR of the investigated fungal isolates was recorded at 410 nm. These findings are partially inconsistent with [12, 39, 57] who mentioned that the silver nanoparticles showed SPR in a range from 350 nm to 435 nm. The position of the SPR peak was found to be varied based on the physicochemical properties of the metal nanoparticles including size, shape, stabilizing and capping agents [16, 39]. Among the tested fungi, the biosynthetic SNPs obtained from the *F. oxysporum* EG-AR3 extract were used for further work based on the absence of agglomerations and precipitation, maximum Uv-vis absorption peak (3.1 au), homogeneity, and high stability. The fungal extract showed an absorption peak at approximately 380 nm which may be related to the presence of various exometabolites in the filtrate, while the negative control (colorless AgNO_3_) had no noticeable peak in the spectrum (Fig. 4A). The XRD analysis for the SNPs synthesized by *F. oxysporum* is illustrated in (Fig. 4B). The XRD pattern displayed four characteristics diffraction peaks at 2Theta = 36.5°, 42.8°, 63.2°, and 76.4° which match the crystallographic plans of Ag (111), (200), (220), and (311), respectively. Similar findings have been described by [58, 59].

The morphological analysis (particle size, distribution, and shape) of the SNPs prepared by harnessing metabolites of *F. oxysporum* was investigated using JEOL JEM-1010 transmission electron microscope (TEM). The developed SNPs were compact, monodispersed, spherical, and irregular in shape with an average particle size of 5 nm as revealed from TEM micrographs (Fig. 4C). The crystalline nature of SNPs was affirmed by the diffraction pattern of the TEM snapshot (Fig. 4D). The green synthesized SNPs exhibited significantly smaller nanoparticle size when compared to the SNPs in literature as clearly illustrated in (Table S3). The biological methods can produce SNPs with controlled particle sizes; however, the physicochemical methods used to produce SNPs with various particle shapes and sizes in the range of 5–100 nm [13, 60, 61]. The absence of SNPs aggregates is due to the outer-layered formed around the particles from bioactive materials which are distributed in the cell-free filtrate during the biosynthetic process [12, 57]. The biosynthetic SNPs derived from exometabolites of *Solibacillus isronensis* (80–120 nm) [62], *Massilia* sp (30–50) [39], and *Alternaria alternate* (66–88 nm) [50] were successfully formed with various particle size. Different applications of SNPs were decidedly dependent on various parameters including their size, crystallographic structure, and shapes. The smaller size of the as-produced nanomaterials predicts a broad possible application in different fields [12, 16, 57]. Hence, the relatively small size of the produced SNPs using exo-metabolites of *F. oxysporum* can predict a promising high activity in different applications.

**Fig. 5:**
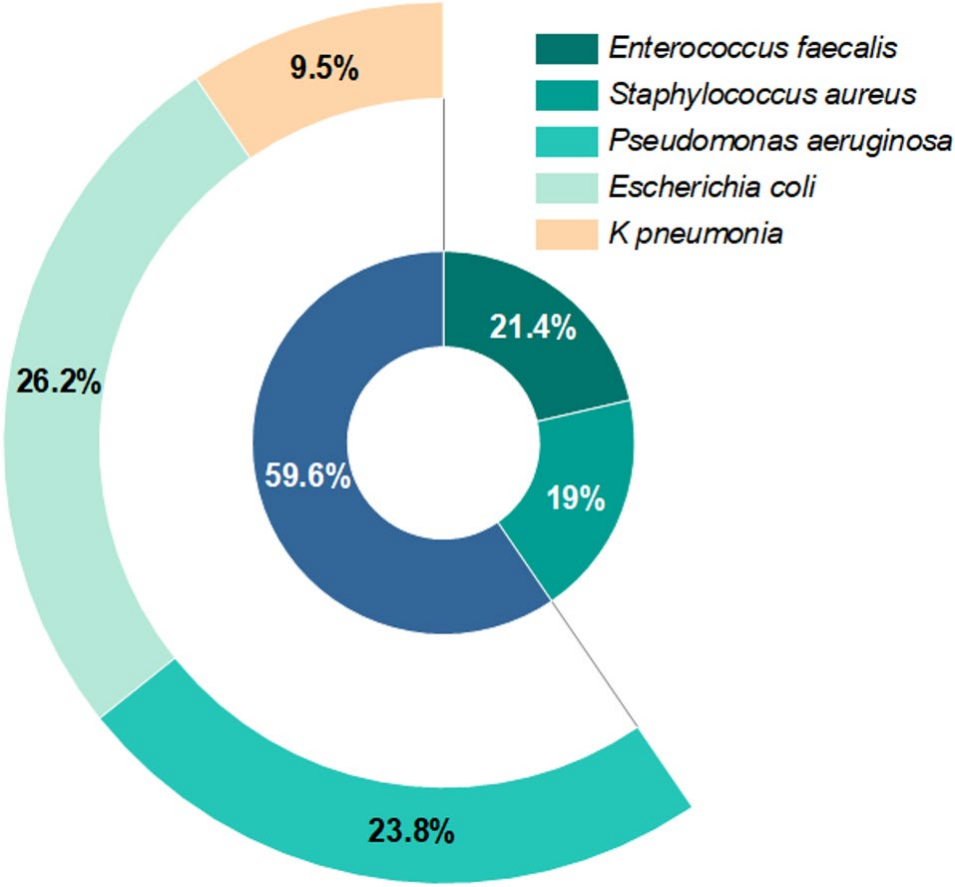
Incidence of different bacterial isolates of the uropathogenic specimens (*n* = 42)

FTIR spectra of fungal filtrate and SNPs arbitrated compound designate that main absorption peaks were detected at 3498 cm^− 1^ (O–H stretching vibration), 2965 cm^− 1^ (C–H or O–H stretching vibration), 1730 cm^− 1^ (C = C or C = O stretching vibration), and 1415 cm^− 1^ (C–O bending vibration) (Fig. 5E). These results specify that the carboxyl and hydroxyl groups from fungal filtrate are accountable for the silver ions bio-reduction and act as coating and capping mediators for the development of functionalized SNPs arbitrated nanoparticles. The water-soluble extracted biomolecules incorporated in the green synthesis of SNPs have been reported to act as a stabilizing, capping, dipping, and coating agent [39, 52].

### Distribution of bacterial isolates and antibiotic susceptibility test

Forty-two bacterial isolates were recovered from the patient’s urine specimens. Based on the morphological characteristics of the isolated bacteria, 25 (59.5%) Gram-negative bacilli and 17 (40.5%) Gram-positive cocci (*Enterococcus faecalis* and *Staphylococcus aureus*) were isolated (Fig. 5). The bacilli groups were found to be *Escherichia coli*, *Pseudomonas aeruginosa*, and *Klebsiella pneumonia*. The antibiograms of these bacterial isolates were determined using twelve antibiotic discs. The results presented in Table S4 illustrated that *Enterococcus faecalis* were mainly sensitive to SAM, FEP, FOX, LEV, and CIP (67%), followed by AK and AM (33%), but mainly resistant to CTX, G and AMC (100%). However, *S. aureus* was mainly too resistant to CTX, SAM, AMC, and AK (100%), followed by FEP, G and CXM and sensitive to AZM (33%), LEV, FOX, and CIP. *P. aeruginosa* was sensitive to CTX, G, AMC, FOX (100%), followed by AZM, CIP (60%), and AK (40%), while *E. coli* was highly resistant to FOX, G, AMC, FOX, and SAM (100%). However, *K. pneumonia* is moderately sensitive to AZM, FEP, CXM, IPM, FOX, LEV, and CIP. The antibiotic susceptibility analysis generally exhibited fluctuations among the isolated uropathogenic bacterial flora, indicating the presence of multi-drug resistance bacteria (MDR). Multiple antibiotic resistance index (MARI) was recorded in the range of 0.42 to 0.67. Hence, three representative isolates that were more resistant to multiple antibiotics (MDR) were selected for further work.

### Molecular identification of the multidrug-resistant bacteria

**Fig. 6:**
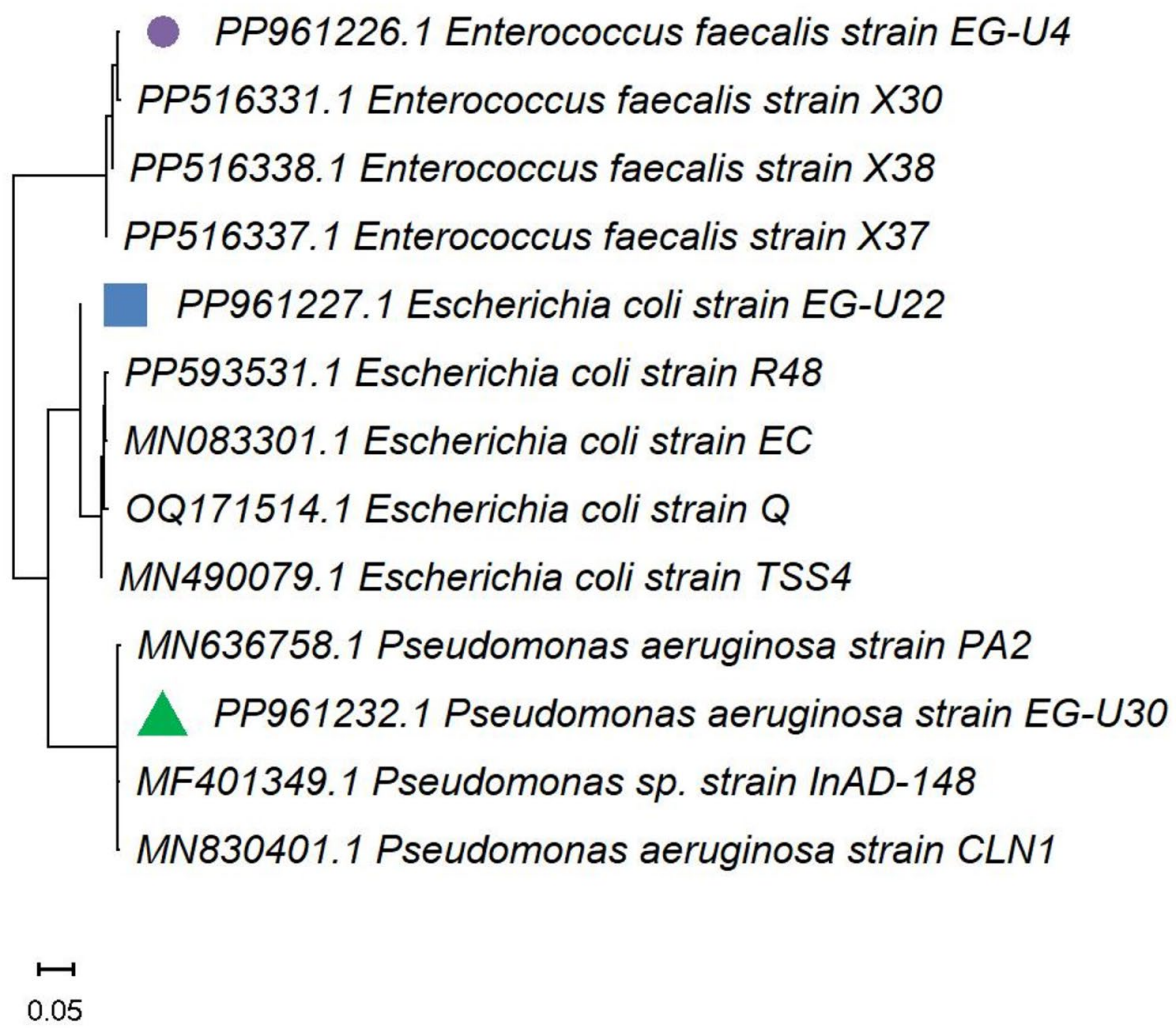
(**A**) Phylogenetic analyses of the multi-drug resistant bacteria based on the amplification and sequencing of 16 S rRNA region. Phylogenetic tree was performed with the NCBI closely related sequences using MEGA-X 11 via the Maximum Likelihood method and default settings. The MDR isolates under study were indicated by symbols. The bar length denotes 0.05 substitutions for each nucleotide site

The 16 S rRNA gene sequencing was used to identify selected three MDR bacterial isolates. The amplicons for EG-U4, EG-U22, and EG-U30 were purified, sequenced, and undergo a non-redundant BLAST search. The partially sequenced 16 S rRNA genes were deposited in the GenBank database and were ascribed to the accession numbers PP961226.1, PP961227.1, and PP961232.1. Phylogenetic trees were constructed with closely related 16 S rRNA sequences on the GenBank (Fig. 6).

### Susceptibility test of the multidrug-resistant bacteria towards SNPs

**Fig. 7:**
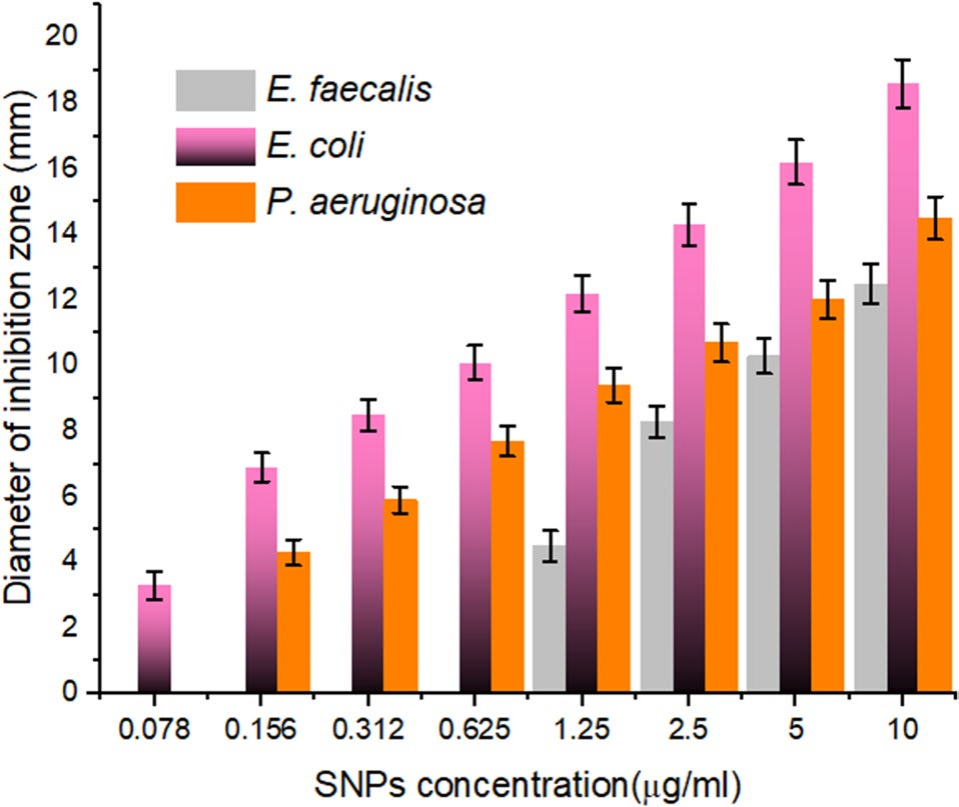
Antibacterial activity (mm) of the SNPs synthesized using the cell free extract of *F. oxysporum* against the tested multi-drug resistant uropathogenic bacteria

The antimicrobial activity of the SNPs synthesized using *F. oxysporum* cell extract was assessed against three multidrug resistant bacteria represented by one Gram-positive (*E. faecalis*) and two Gram-negative (*P. aeruginosa* and *E. coli*) as shown in Fig. 7. The evaluation process is based on the correlation between the concentration of SNPs and the corresponding inhibition zones. The results showed a dramatic reduction of bacterial growth using different SNPs concentrations. The antibacterial action of the biosynthetic SNPs was found to be concentration-dependent. The SNPs synthesized using *F. oxysporum* showed higher inhibitory zones when compared with the results obtained by [30] who found that the antibacterial activity of SNPs was in a range of 7–20 mm when investigated against *B. subtilis*, *S. aureus*, *E. coil*, and *Pasteurella multocida*. Moreover [6], observed that the inhibitory zones were in the range of 2 to 18 mm using biogenic SNPs against *S. aureus*, *B. subtilis*, *E. coil*, and *Salmonella typhi*. Similar findings have been reported by investigators who proved the dose-dependent behavior of the antibacterial activity of different nanoparticles [12, 13]. The fluctuations in the diameters of inhibition zones on plates as a function of the concentration of biosynthetic SNPs are possibly attributed to the variation in the bacterial cell composition, physiology, charge, and metabolism as well as the potential charge of the investigated SNPs. It is suggested that SNPs induce the production of various free radicals and leakage of proteins and polysaccharides from the cell, resulting in the breakdown of the proton motive force and membrane potential [2, 13, 39]. In the current study, the strong antibacterial activity of the green synthesized SNPs may be attributed to their smaller size which permits them to easily attach and diffuse into the cells, compared to the larger SNPs. The spherical shape of the SNPs performed the largest available surface area to interact with the bacterial pathogens.

### Determination of MIC and MBC

The bacteriostatic and bactericidal effect of the developed SNPs was assessed based on the inhibition effect of serial doubling dilutions of the nanoparticles on the bacterial growth, growing in MH broth. The MIC values of the green synthetic SNPs towards the investigated uropathogenic bacteria were found to be in a range from 0.078 µg/ml to 1.25 µg/ml, while the MBC values ranged from 0.156 to 0.125 µg/ml (Table 3). The MIC and MBC of the green SNPs towards *S. faecalis* was 1.25 µg/ml. The MIC values of the SNPs against *P. aeruginosa* and *E. coli* were respectively found to be 0.156 µg/ml and 0.078 µg/ml, while the MBC were 0.312 µg/ml and 0.156 µg/l. The values of MIC and MBC revealed that *E. faecalis* was less susceptible to SNPs while *E. coli* displayed a lower MBC value, compared to the other tested pathogenic bacteria. Large fluctuations in the MIC values have been recorded by several researchers as there is no typical method for assessing the antibacterial effect of SNPs. Hence, it is difficult to compare the obtained results with the previous ones obtained by research groups [6, 59].

**Table 3:** Results of the minimum inhibitory concentration and minimum bactericidal concentration (µg/l) of the biosynthesized silver nanoparticles using *F. oxysporum*

Bacterial strain	SNPs (µg/ml)	
	MIC	MBC
*E. faecalis*	1.25	1.25
*E. coli*	0.078	0.156
*P. aeruginosa*	0.156	0.312

* The MIC and MBC of the biosynthetic SNPs were determined by testing the influence of serial doubling dilutions (starting at 10 µg/ml) on the bacterial growth

### Time-kill kinetics of SNPs

**Fig. 8:**
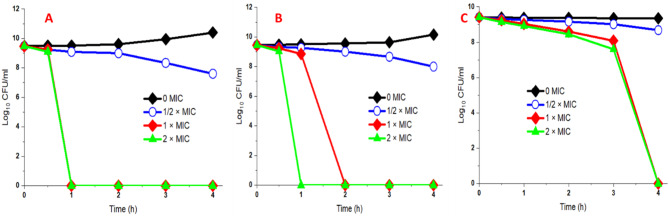
Time-course kinetics curve plots of the antibacterial activity of the biosynthetic silver nanoparticles using *F. oxysporum* extract against *E. coli* (**A**), *P. aeruginosa* (**B**), and *E. faecalis* (**C**). All bacteria were examined against the 0 × MIC, 1/2 × MIC, 1 × MIC, and 2 × MIC. Aliquots (100 µl) were withdrawn at time intervals (0, 0.5, 1, 2, 3 and 4 h), plated onto Muller-Hinton agar and the viability was expressed in Log_10_ cfu/ml

A kill-time analysis assay was performed using the green SNPs at three various concentrations: 0 × MIC, ½ × MIC, 1 × MIC, and 2 × MIC values. A dramatic reduction in the killing rate for all investigated uropathogenic bacteria tested in the presence of the green synthesized SNPs over 4 h of incubation was determined as illustrated in Fig. 8A-C. The biosynthetic SNPs evaluated at a concentration of ½ × MIC displayed a bacteriostatic effect on the tested pathogenic bacteria when plotting the log_10_ cfu/ml versus time. The bactericidal endpoint of SNPs for *E. coli* was accomplished after 1 h of incubation at 1 × MIC (0.078 µg/ml) and 2 × MIC (0.156 µg/ml) (Fig. 8A); while the *P. aeruginosa* exhibited a bactericidal effect when tested at a concentration of 1 × MIC (0.156 µg/ml) for 2 h and 2 × MIC (0.312 µg/ml) for 1 h (Fig. 8B). In the same context, SNPs were evaluated for *E. faecalis* at a concentration of 1.0 × MIC (1.25 µg/ml) and 2 × MIC (2.5 µg/ml) displayed a killing effect after 4 h of incubation (Fig. 8C). A considerable and approximately analogous reduction and killing rate of *P. aeruginosa* and *E. faecalis* has been observed as the log_10_ of cfu/ml concentration with incubation time continued approximately at the same trend; however, the highest reduction was recorded for *E. coli* when the bactericidal effect of SNPs was evaluated.

In the time-kill assay, the bacteriostatic and bactericidal effects of the biosynthetic SNPs were found to be in a time and dose-dependent manner. The results also reveal that the biosynthetic SNPs showed a broad-spectrum antimicrobial agent as they displayed a similar effect for both Gram-positive and negative uropathogenic bacteria. The bacterial cell viability of the urophathogenic *E. coli* was decreased by increasing contact time with the biogenic silver nanoparticles [11, 24]. The viability of bacterial cells was dose-dependent, related to different SNPs concentrations. The complete kill of *E. coli* treated with MIC of SNPs was recorded after 1 h of incubation. These findings are in consistent with [63].

Resazurin dye has been utilized in the bacterial growth determination. Viable cells-oxidoreductases can reduce resazurin indicator (non-fluorescent blue) to resorufin (fluorescent pink) [58, 64]. In this study, the biosynthetic SNPs are considered excellent antibacterial agents that completely kill a high bacterial concentration of roughly 10^6^ cfu/ml, which infrequently existed in real-life systems. These results also proved that the obtained SNPs synthesized by *F. oxysporum* extract were able to kill all investigated bacteria at low concentrations in a shorter contact time. The bactericidal activity of SNPs against MDR bacteria may be attributed to the unique physical and chemical properties as well as the high surface area-to-volume ratio which increases with reducing the particle sizes during the synthesis process. The smaller the SNPs size, the easier adherence and penetration into the microbial cell. However, the exact mechanism of the antibacterial effect of SNPs remains anonymous. It is proposed that the SNPs induce the production of various free radicals, inactivation, and leakage of proteins and polysaccharides from the cell, resulting in the breakdown of the proton motive force and membrane potential [25, 58, 65].

### Morphological observation

**Fig. 9:**
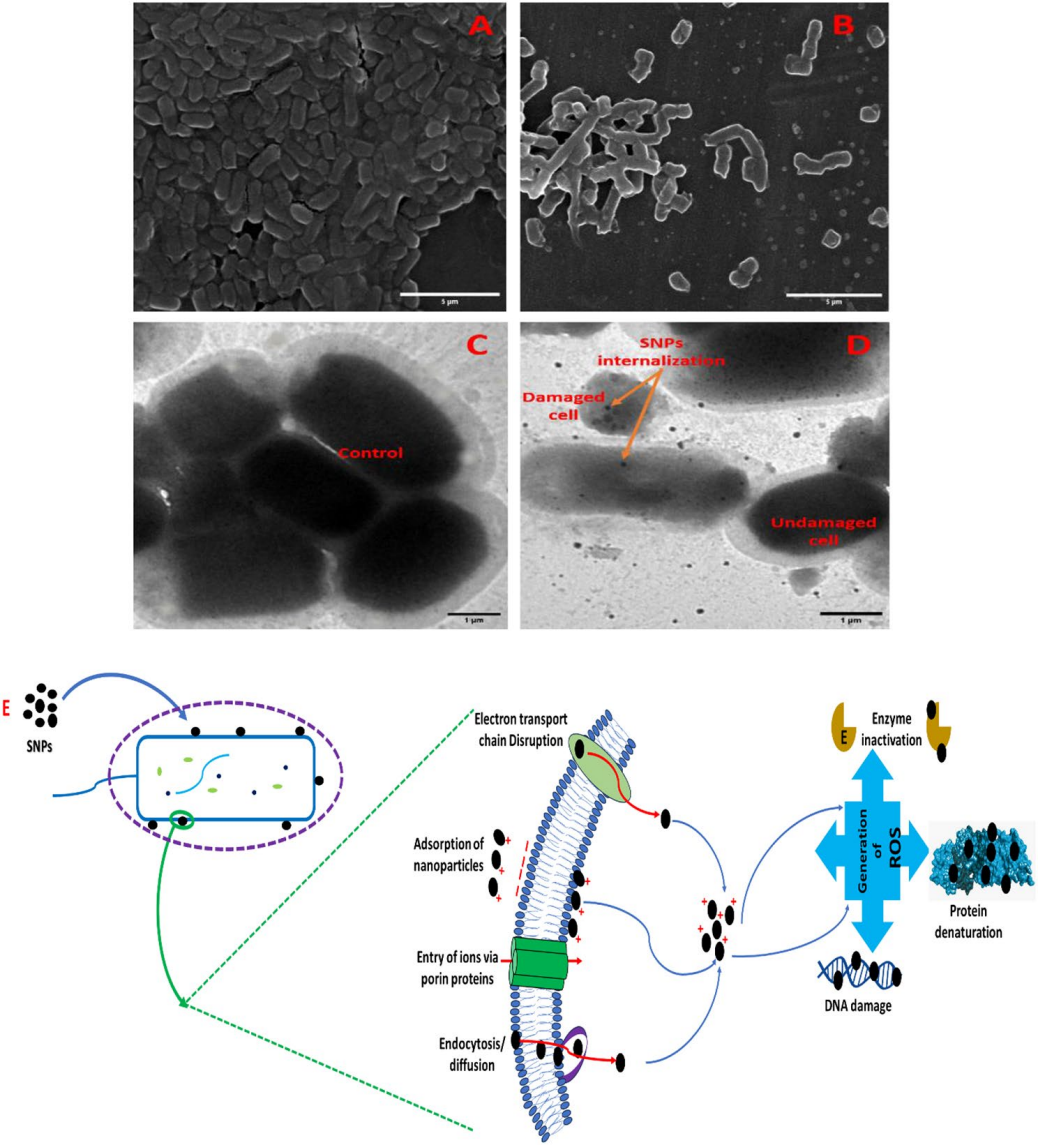
Representative SEM images showing the induction of SNPs synthesized by harnessing biomolecules in *F. oxysporum* extract on *E. coli* for 1 h. Control (**A**) represents the morphology of the bacteria without SNPs, while *E. coli* treated with SNPs (**B**). Magnification is 5,000× and scale bar represents 5 μm. TEM images of (**C**) control *E. coli* (overnight old) and (**D**) *E. coli* treated with SNPs. Scale bar is 1 μm

The morphological alterations in bacterial cells on exposure to mycosynthesized SNPs were determined using SEM (Fig. 9A, B) and TEM (Fig. 9C, D). Based on the MIC assay, *E. coli* has been selected for this experiment as a more susceptible bacteria for SNPs. The MBC dose (0.156 µg/ml) was supplemented with the bacterial broth culture. The bacterial culture was examined using SEM, relating to control (deprived of SNPs, Fig. 9A). Distortion of bacterial cells, cytoplasmic leakage, and significant reduction in the cells of *E. coli* were detected on exposure to SNPs (Fig. 9B). TEM micrograph was performed to visualize the changes in the ultracellular structure of *E. coli* and the internalization of SNPs in the bacteria cells, compared to negative control (deprived of SNPs, Fig. 9C). The internalization of SNPs inside the cellular structure of the tested bacterium has been noticed (Fig. 9D). On exposure to SNPs, a dramatic alteration in the bacterial cell structure, distortion in the cell wall, and leakage of cellular contents was remarkably determined as shown in Fig. 9D.

The positively charged SNPs initiate an electrostatic interaction with the negatively charged bacterial cell, subsequently adhere, and diffuse inside the bacterial cell. The formation of several pits in the bacterial cell wall and structure disruption have occurred due to the interaction between silver ions and phosphorus and sulfur-containing biomolecules in the cell wall of bacteria. These small cavities affect the integrity of the cell wall and permit the influx of foreign materials, causing a rise in the intracellular osmotic pressure. The bacterial cell then swells, followed by cell wall rupture and finally cell lysis [66]. The SNPs can adhere to the bacterial cell membrane, interfering with lipopolysaccharides, lipids, and proteins. They can generate reactive oxygen species, which can damage the electron transport chain, and bacteria’s protein, mitochondria, and DNA. The proposed schematic representation of the biosynthetic SNPs is clearly illustrated in Fig. 9E. The results are in convincing agreement with that obtained for different Gram-negative bacteria on treatment with SNPs and monitored at the microscopic level [25, 58, 562, 65]. The antimicrobial activity of the green synthesized SNPs is related to the physical attrition of the bacterial cells with various nanoparticles [25, 67]. A remarkable cracking in the bacterial cell borders and pores formation are visible on treating the bacteria with SNPs, leading to the release of the internal cytoplasmic contents [25, 59, 65, 67].

### Antioxidant activity of SNPs

**Fig. 10:**
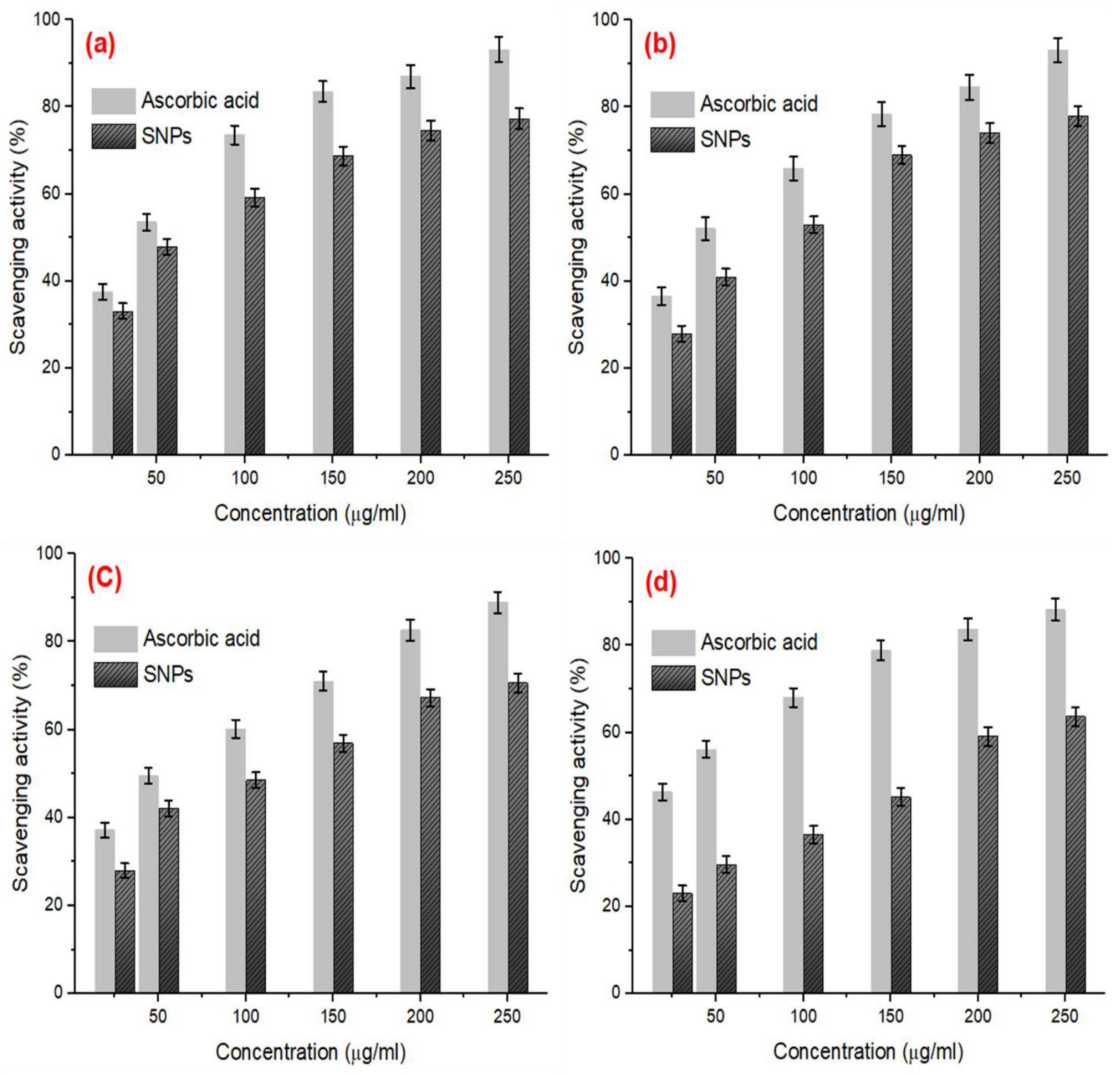
(**a**) DPPH, (**b**) superoxide, (**c**) hydrogen peroxide, and (**d**) hydroxyl radicals scavenging activity of the biosynthetic SNPs using ascorbic acid as reference standard

The antioxidant properties of biosynthetic SNPs were investigated using DPPH, superoxide, hydrogen peroxide, and hydroxyl radical as illustrated in Fig. 10. The DPPH scavenging ability of SNPs exhibited a dose-dependent concentration pattern; however, the antioxidant activity of the investigated sample was quite lower than that for ascorbic acid as a standard reference (Fig. 10a). The IC_50_ of SNPs and ascorbic acid were 74.3 and 37.3 µg/ml, respectively.

The superoxide radical scavenging potentiality of SNPs and ascorbic acid was illustrated in Fig. 10b. A concentration-dependent manner has been observed during the investigation of SNPs scavenged ability of superoxide radical. The rise in the concentration of SNPs revealed a remarkable increase in the inhibition percentage of superoxide radicals.

The hydrogen peroxide scavenging ability of SNPs was found to be increased correspondingly with the elevation in the sample concentration (Fig. 10c). At the highest concentration (250 µg/ml), the maximum inhibition of H_2_O_2_ radical scavenging ability was approximately 70.6% using SNPs, compared with the activity of ascorbic acid (88.9%).

The antioxidant property of SNPs was also evaluated by determining the scavenging ability toward hydroxyl radicals as depicted in Fig. 10d. The OH-scavenging ability of SNPs was remarkably increased with increasing the sample concentration, while the maximum inhibition was 63.7% and 88.2% at 250 µg/ml for SNPs and ascorbic acid, respectively.

Based on the above results, the antioxidant properties of SNPs using DPPH, superoxide, hydrogen peroxide, and hydroxyl radical were relatively higher over the SNPs derived using various biological sources [43, 68, 69]. Additionally, the antioxidant activity of SNPs displayed an IC_50_ values of 203.67, 146.58, 131.87, and 187.77 µg/ml on ABTS, DPPH, H_2_O_2_, and OH, respectively [47]. Similar results have been earlier described for the scavenging ability of silver nanoparticles [9, 69–71]. The antioxidant properties of SNPs have been investigated by other researchers using DPPH, superoxide, hydrogen peroxide, nitric oxide, and hydroxyl radicals [69, 67]. The scavenging capability of antioxidants is based on the hydrogen donation to the DPPH radical [70]. The superoxide radical is one of the reactive oxygen species which is mainly responsible for cellular damage [43, 70]. The hydrogen peroxide radical is a major injurious to cellular materials and energy-generating systems. The hydroxyl radical is principally caused by DNA damage and protein peroxidation [72]. The greater antioxidant ability of the green synthesized SNPs is mostly due to the presence of capping material which is composed of the bioactive compounds in the developed filtrate [9, 69].

### Cytotoxic effect of SNPs

**Fig. 11:**
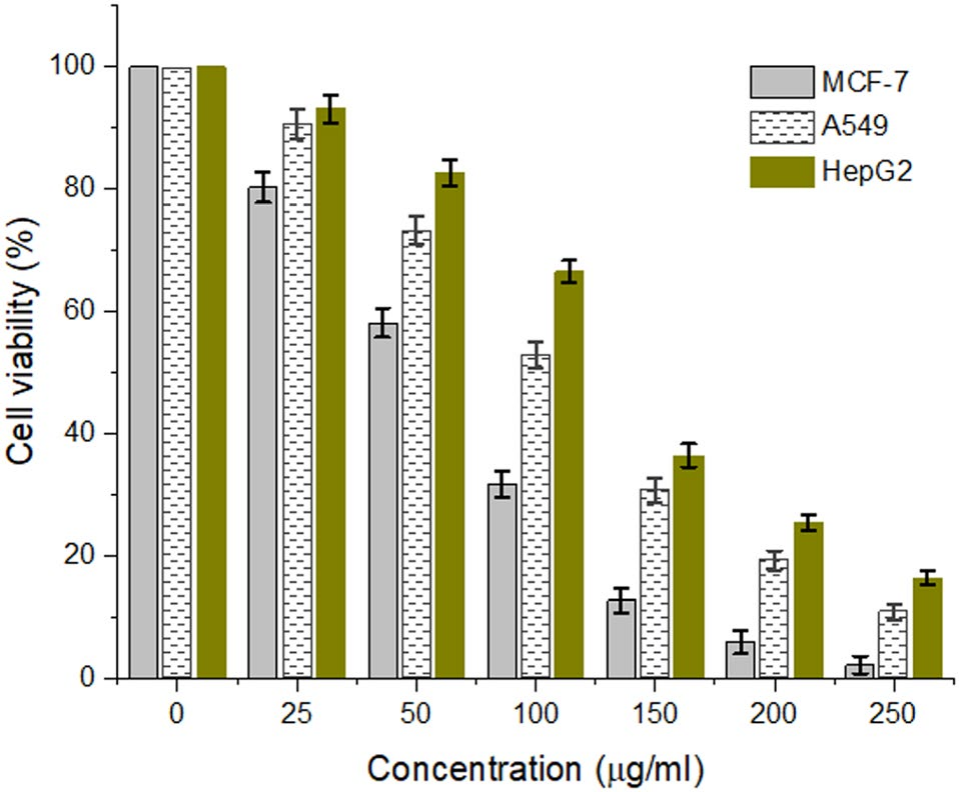
Cytotoxic efficiency of biosynthesized SNPs using MTT assay. Different concentrations of tested samples were incubated with the investigated cells at 37 °C for 3 h

The cytotoxic behavior of the biosynthesized SNPs was evaluated against different cell lines namely, MCF-7, A549, and HepG-2 cell carcinoma, and the cell viability percentage was illustrated in Fig. 11. The cell viability percentage was found to be reduced with increasing the SNPs concentration; however, the cytotoxic activity was increased in the investigated cell lines during the rise in the concentration of SNPs. The inhibitory concentration (IC_50_) values against MCF-7, A549, and HepG-2 cells were correspondingly found to be 89.4, 121.4, and 138.9 µg/ml. The results showed that the most susceptible cell for SNPs in the present study was MCF-7 cells, compared with other tested cell lines. Hence, the MCF-7 cells were further examined using an inverted microscope as illustrated by Figure S1. The IC_50_ of MCF-7, and HepG2 treated with SNPs synthesized by *F. nygamai* were found to be 302.93, and 309.98, respectively [47]. Similar findings are reported for the anticancer activity of the green synthesized SNPs versus different cell carcinoma [14, 17, 47]. The inhibitory activity of SNPs is largely dependent on the morphology, size, and bioactive capping compounds surrounding nanoparticles [73]. The precise mechanism of cytotoxic activity of SNPs is not yet described, however, it is suggested that the anticancer activity of biosynthesized SNPs may be attributed to the ability of SNPs to generate reactive oxygen species, induce cancer cells apoptosis via mitochondrial- and caspase-dependent pathways [21, 30, 73]. The SNPs exposure induces oxidation stress and cytotoxicity. SNPs can disrupt the cell membrane integrity and rupture the lysosomal membrane. SNPs can directly reduce cell viability, modify cell shape, trigger free radicals’ production, increase release of dehydrogenase and lipid peroxidation, reduce superoxide dismutase and glutathione, inhibit synthesis of adenosine triphosphate (ATP), increase DNA fragmentation and caspase-3 activity which eventually leads to cell apoptosis occurred [74]. Once SNPs are internalized into mitochondria, they can impair cell membranes, accelerate oxidative stress, repose the cell cycle, aberrate chromosomes, damage DNA, and apoptosis.

### Crystal violet- decolorization using SNPs

**Fig. 12:**
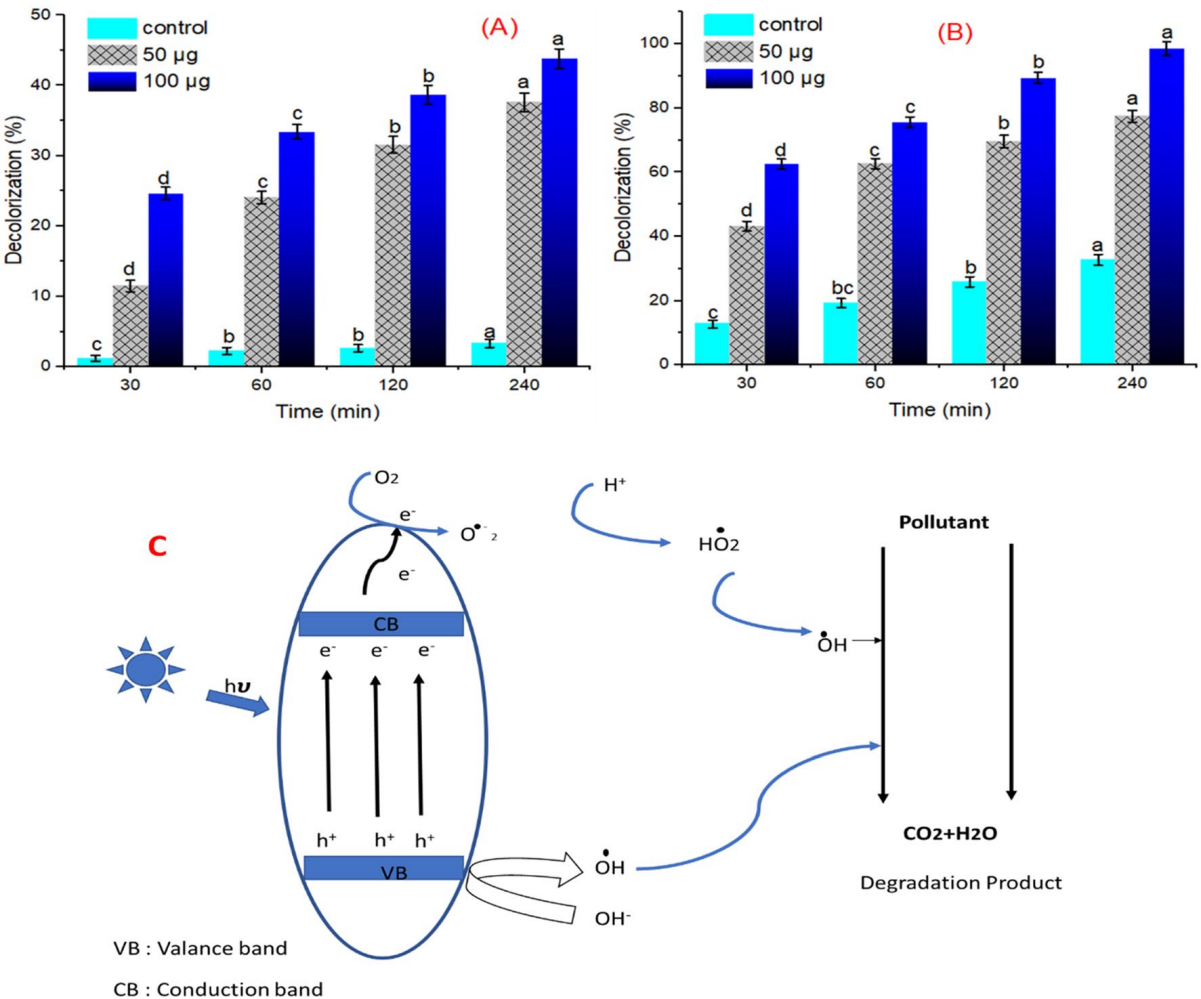
Photocatalytic decolorization (%) using various concentrations of SNPs for crystal violet dye degradation at various contact times and in various stimulation conditions under dark (**A**) and light irradiation (**B**). Data of crystal violet dye decolorization (%) under dark (red line) and light irradiation (blue line) are represented as mean ± standard deviation (*n* = 3). Data are significantly distinct at *P* < 0.05 by Tukey’s HDS test and are presented as different letters with the same line color. (**C**) Possible decolorization mechanism of crystal violet dye using the green synthesized SNPs

The photocatalytic potentiality of the biosynthetic SNPs in the current study was investigated by assessing the crystal violet degradation ability at various contact times in dark and light simulations. Figure 12A&B clearly showed that the degradation activity of CrV dye at 100 mg/l using SNPs in dark and light irradiation was performed in a time- and concentration-dependent manner. The smaller-sized SNPs obtained using *F. oxysporum* can highly decolorized CrV dye. In the dark, the decolorization efficiency of 33.40 ± 0.3% after 60 min was determined at low SNPs concentration (100 µg), while the decolorization efficiency under light irradiation was found to be 75.60 ± 0.5% after 60 min was determined. The highest decolorization percentage of 98.60 ± 0.7 was detected after 240 min at 100 µg SNPs concentration under the light source. SNPs were produced with an average size of 27.3–53.1 nm using *Fusarium nygamai* which displayed decolorization percentages after 240 min of 88.3, 76.4, 81.5, and 78.2 for methylene blue, crystal violet, safranin, and green malachite, respectively [47]. Compared with the previous data, the SNPs of *F. oxysporum* showed a superior decolorization (%), hinting a promising application in dye decolorization. The interaction of water molecules and semiconductor nanomaterial during photocatalytic reaction produces reactive $\:{\text{O}}_{2\:}^{{\bullet\:}-}\:$and OH^•^ which initiates and increases the degradation of the dye with minimal toxicity as shown by. Figure 12C and the following equation:

6 $$\mathrm{CrV}+\mathrm{O}_2^{\bullet-}+\mathrm{OH} \bullet \mathop{\longrightarrow}\limits^{\text{nanocatalyst}} \mathrm{H}_2 \mathrm{O}+\mathrm{CO}_2+\text { degrading products }$$

ROS plays a vital role in the decomposition of organic contaminants through redox reactions during photocatalytic reactions. They are produced when electron pairs react with H_2_O and O_2_ molecules sorbed onto the photocatalyst’s surface. The photogenerated electron (e^−^) is the main factor in the catalytic performance. The photogenerated holes will react with water molecules sorbed on the photocatalytic surface, producing OH^•^. Several electrons reduce the sorbed O_2_ on the surface, yielding $\:{\text{O}}_{2}^{{\bullet\:}-}$. These ROS (OH^•^ and $\:{\text{O}}_{2}^{{\bullet\:}-}$) will sharply decompose the organic dye in the reaction mixture [75].

## Conclusion

This study provides an effective and eco-friendly approach for producing small-sized silver nanoparticles using a supernatant of filamentous fungi. Statistical optimization of SNPs conditions using Placket Buran and central composite design verified the ability to produce SNPs with a very small-sized, and highly abundant amount. The maximum biosynthesis was achieved using the subsequent factors: pH (5), temperature (20 °C), metal precursor (2 mM), and biomass amount (5 g). The developed bio-nano-silver displayed not only remarkable in vitro antioxidant and anticancer activities but also a significant killing rate on different multidrug-resistant bacteria, exhibiting a great potential application in various therapeutic fields. The biogenic SNPs exhibited high decolorization efficiency of organic dye contaminants under light irradiation, hinting at their potential application in photocatalysis. The biosynthetic process has certain drawbacks including the selection of suitable microbial supernatant, the nature and quality of biomolecules found in microbial supernatant for SNPs synthesis, and the strong fluctuation in the size, charge, and shape of SNPs. To scale-up the biosynthesis process, further investigations are required to exactly identify and extract proteins responsible for SNPs biosynthesis or artificially producing the identified proteins. Bioassay standardization can perform reproducible and reliable data for comparing the effect of SNPs on various cells. Finally, the applications of biosynthetic SNPs may provide valuable conclusions in different fields, particularly antimicrobial systems, dye bioremediation, and medical devices.

## Electronic supplementary material

Below is the link to the electronic supplementary material.

Supplementary Material 1

## Data Availability

Sequence data that support the findings of the current study have been deposited in the NCBI (National Biotechnology Information Center) nucleotide database with the GenBank accession numbers of PP961238.1, PP961226.1, PP961227.1, and PP961232.1.

## Abbreviations

SNPs: Silver Nanoparticles
MDR: Multidrug Resistant
DPPH: 2,2-Diphenyl-1-Picrylhydrazyl
PBD: Plackett-Burman Design
CCD: Central Composite Design
MBCs: Minimum Bactericidal Concentrations
MICs: Minimum Inhibitory Concentrations
RSM: Response Surface Methodology
MHA: Muller-Hinton Agar
