# Supplementary material for: Fusarium oxysporum assisted green synthesis of small-sized silver nanoparticles for high antibacterial, and photocatalytic decolorization performances
Source: BMC Microbiol. 2025 Jan 6;25:4. doi: 10.1186/s12866-024-03686-7 (PMC11702280; doi:10.1186/s12866-024-03686-7)
Supplement: Supplementary file 1 — Supplementary Material 1 [file 12866_2024_3686_MOESM1_ESM.docx]

***BMC Microbiology***

***Fusarium oxysporum* assisted green synthesis of small-sized silver nanoparticles for high antibacterial, and photocatalytic decolorization performances**

**Reyad M. El-Sharkawy^1*^, Inas A. Ahmed^2,3^, Taghrid G. Kharboush^4^**

*^1^Botany and Microbiology Department, Faculty of Science, Benha University, Egypt,* [r.m.elsharkawy@fsc.bu.edu.eg](mailto:r.m.elsharkawy@fsc.bu.edu.eg)*.*

*^2^Department of Medical Biochemistry and Molecular Biology, Faculty of Medicine, Benha University, Egypt,* [*inas.ahmed@fmed.bu.edu.eg*](mailto:inas.ahmed@fmed.bu.edu.eg)*.*

*^3^Central Laboratory for Research, Faculty of Medicine, Benha University, Egypt.*

*^4^Department of Medical Microbiology and Immunology, Faculty of Medicine, Benha University, Egypt,* [*t.g.kharboush@gmail.com*](mailto:t.g.kharboush@gmail.com)*.*

*Correspondence: [r.m.elsharkawy@fsc.bu.edu.eg](mailto:r.m.elsharkawy@fsc.bu.edu.eg), <http://orcid.org/0000-0003-1319-9066>

**Table S1**: 16-trial experimental design of Plackett Burman for the SNPs production using the filtrate of *F. oxysporum*. The signs “+1′′ and “+1′′ represent the high and low level of the input variables.

| Run | Blocks | Parameters | | | | | | Response | | |
| --- | --- | --- | --- | --- | --- | --- | --- | --- | --- | --- |
|  |  | pH | Temperature | Time | Ag precursor concentration | Biomass amount | Ratio | Actual | Predicted | Residual |
| 1 | 1 | 5 | 20 | 60 | 2 | 5 | -1 | 2.98 | 2.995 | -0.015 |
| 2 | 1 | 10 | 20 | 60 | 0.5 | 10 | -1 | 2.11 | 2.500 | -0.390 |
| 3 | 1 | 10 | 50 | 0 | 2 | 5 | -1 | 1.89 | 1.815 | 0.075 |
| 4 | 1 | 5 | 20 | 60 | 2 | 5 | -1 | 3.01 | 2.995 | 0.015 |
| 5 | 1 | 5 | 50 | 0 | 0.5 | 10 | -1 | 2.01 | 2.190 | -0.180 |
| 6 | 1 | 10 | 20 | 60 | 0.5 | 10 | -1 | 2.89 | 2.500 | 0.390 |
| 7 | 1 | 5 | 20 | 0 | 2 | 10 | 1 | 2.05 | 2.100 | -0.050 |
| 8 | 1 | 10 | 50 | 0 | 2 | 5 | -1 | 1.74 | 1.815 | -0.075 |
| 9 | 1 | 10 | 20 | 0 | 0.5 | 5 | 1 | 2.77 | 2.825 | -0.055 |
| 10 | 1 | 5 | 50 | 60 | 0.5 | 5 | 1 | 2.61 | 2.785 | -0.175 |
| 11 | 1 | 10 | 50 | 60 | 2 | 10 | 1 | 1.36 | 1.170 | 0.190 |
| 12 | 1 | 10 | 50 | 60 | 2 | 10 | 1 | 0.98 | 1.170 | -0.190 |
| 13 | 1 | 5 | 50 | 60 | 0.5 | 5 | 1 | 2.96 | 2.785 | 0.175 |
| 14 | 1 | 10 | 20 | 0 | 0.5 | 5 | 1 | 2.88 | 2.825 | 0.055 |
| 15 | 1 | 5 | 50 | 0 | 0.5 | 10 | -1 | 2.37 | 2.190 | 0.180 |
| 16 | 1 | 5 | 20 | 0 | 2 | 10 | 1 | 2.15 | 2.100 | 0.050 |

**Table S 2:** 25-trial experimental design of CCD for the SNPs production using the filtrate of *F. oxysporum*. The sign of “+1′′ and “+1′′ represent the high and low level of the input variables

| Blocks | Parameters | | | | Response | | |
| --- | --- | --- | --- | --- | --- | --- | --- |
|  | pH | Temperature | Ag precursor concentration | Biomass amount | Actual | Predicted | Residual |
| 1 | 5 | 20 | 0.5 | 5 | 2.03 | 2.19 | -0.16 |
| 1 | 10 | 20 | 0.5 | 5 | 1.69 | 1.62 | 0.07 |
| 1 | 5 | 50 | 0.5 | 5 | 1.14 | 0.83 | 0.31 |
| 1 | 10 | 50 | 0.5 | 5 | 1.08 | 0.62 | 0.46 |
| 1 | 5 | 20 | 2 | 5 | 3.48 | 3.05 | 0.43 |
| 1 | 10 | 20 | 2 | 5 | 2.16 | 2.18 | -0.02 |
| 1 | 5 | 50 | 2 | 5 | 1.99 | 1.95 | 0.04 |
| 1 | 10 | 50 | 2 | 5 | 0.99 | 1.43 | -0.44 |
| 1 | 5 | 20 | 0.5 | 10 | 2.75 | 1.93 | 0.82 |
| 1 | 10 | 20 | 0.5 | 10 | 1.12 | 1.12 | 0.00 |
| 1 | 5 | 50 | 0.5 | 10 | 1.11 | 1.05 | 0.06 |
| 1 | 10 | 50 | 0.5 | 10 | 0.55 | 0.59 | -0.04 |
| 1 | 5 | 20 | 2 | 10 | 1.65 | 2.08 | -0.43 |
| 1 | 10 | 20 | 2 | 10 | 1.04 | 0.97 | 0.08 |
| 1 | 5 | 50 | 2 | 10 | 1.77 | 1.45 | 0.32 |
| 1 | 10 | 50 | 2 | 10 | 0.89 | 0.69 | 0.20 |
| 1 | 2.5 | 35 | 1.25 | 7.5 | 1.52 | 2.00 | -0.48 |
| 1 | 12.5 | 35 | 1.25 | 7.5 | 0.74 | 0.68 | 0.06 |
| 1 | 7.5 | 5 | 1.25 | 7.5 | 2.03 | 2.21 | -0.18 |
| 1 | 7.5 | 65 | 1.25 | 7.5 | 0.34 | 0.58 | -0.24 |
| 1 | 7.5 | 35 | -0.25 | 7.5 | 0.35 | 0.90 | -0.55 |
| 1 | 7.5 | 35 | 2.75 | 7.5 | 1.99 | 1.86 | 0.13 |
| 1 | 7.5 | 35 | 1.25 | 2.5 | 2.19 | 2.32 | -0.13 |
| 1 | 7.5 | 35 | 1.25 | 12.5 | 1.03 | 1.32 | -0.29 |
| 1 | 7.5 | 35 | 1.25 | 7.5 | 2.08 | 2.08 | 0.00 |

**Table S 3:** Silver nanoparticles fabricated using water extract of different fungi.

| **Microorganisms** | **Uv peak (nm)** | **Size (nm)** | **Shape** | **Reference** |
| --- | --- | --- | --- | --- |
| *Fusarium oxysporum* | 410 | ~ 5 | Monodispersed, spherical | **Current study** |
| *Fusarium oxysporum* | 420 | 25 | Monodispersed, spherical | (Bhainsa and D'souza, 2006) |
| *Fusarium oxysporum* | 420 | 20-33 | Spherical | (Birla et al., 2013) |
| *Trichoderma harzianum* | 430 | 35-50 | Spherical | (Ahluwalia et al., 2014) |
| *Trichoderma harzianum* | 412 | 20 | Irregular | (Sundaravadivelan and Padmanabhan, 2014) |
| *Pseudomonas fluorescens* | 425 | 50 | Polydisperse, myriad | (Syed et al., 2016) |
| *Penicillium oxalicum* | 450 | 32 | Polydisperse, cubic | (Rose et al., 2019) |
| *Aspergillus flavus* | 401 | >35 | Monodispersed, spherical | (Al-Soub et al., 2022) |
| *Glutamicibacter nicotianae* | 416 | 9.67 | Polydisperse, cubic | (Abd El-Ghany et al., 2023) |
| *Fusarium oxysporum* | - | <10 | - | (Chatterjee et al., 2023) |

**Table S 4:** Antibiotic susceptibility profile of *E. faecalis, Staph. aureus*, *E. coli*, *P. aeruginosa* and *K. pneumonia* against twelve antibiotic discs.

|  | **Number of strains** | **CTX** | **AZM** | **P** | **SAM** | **FEP** | **AMC** | **CXM** | **IPM** | **FOX** | **AK** | **LEV** | **CIP** |
| --- | --- | --- | --- | --- | --- | --- | --- | --- | --- | --- | --- | --- | --- |
| *E. faecalis* | 3 | R | S | R | R | I | R | I | R | I | S | I | R |
|  | 6 | R | I | R | S | S | R | S | S | S | R | S | S |
| *Staph. aureus* | 5 | R | R | I | R | I | R | I | S | S | R | R | S |
|  | 3 | R | S | S | R | I | R | I | S | S | R | S | S |
| *E. coli* | 3 | R | I | R | R | I | I | S | S | R | I | R | R |
|  | 4 | R | S | I | R | S | R | I | S | I | S | S | S |
|  | 4 | R | S | S | S | S | R | S | S | I | I | S | S |
| *P. aeruginosa* | 5 | R | R | R | I | S | I | I | S | I | S | S | S |
|  | 5 | R | S | R | I | I | S | I | I | S | R | S | S |
| *K. pneumonia* | 4 | R | S | R | R | S | R | S | S | S | R | R | S |

* cefotaxime (CTX, 30µg), Azithromycin (AZM, 15 µg), penicillin (G, 10µg), ampicillin-sulbactam (SAM), cefepime (FEP, 30µg), amoxicillin-clavulanic acid (AMC, 20+10µg), cefuroxime (CXM), Imipenem (IPM), Cefoxitin (FOX), amikacin (AK, 30µg), levofloxacin (LEV), ciprofloxacin (CIP, 1 µg), R resistant, I intermediate, S sensitive.

**Figure S 1:** Cytotoxic susceptibility of MCF-7 to the biosynthetic SNPs (A) control, (B) Treated.

**
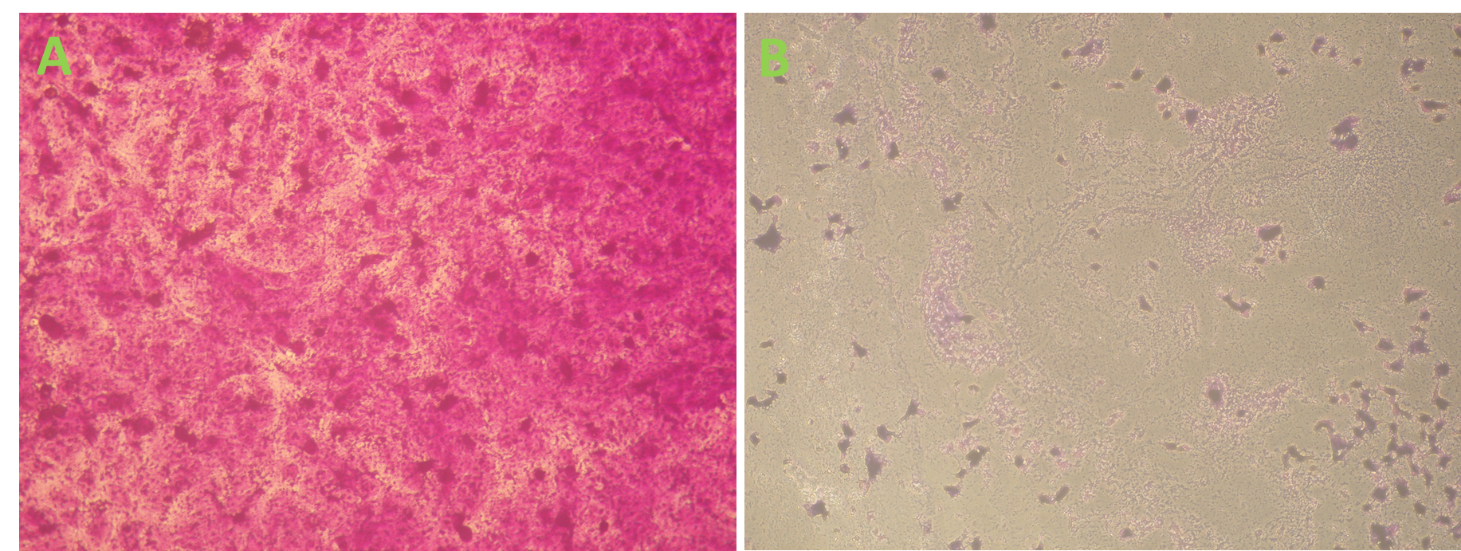
**

**Preparation of Resazurin solution**

Resazurin salt powder was purchased via Sigma-Aldrich. A solution of resazurin (0.03%, w/v) was prepared concurring to the method of Sharaf et al. (Sharaf et al., 2022). Resazurin powder (0.006) was dispersed in distilled water (20 ml) while stirring. Afterwards, the solution was filtered using 0.2 mm membrane filter and can be stored for 15 days at 4 °C.

**Process factors optimization using Plackett-Burman design**

The selected synthesis factors namely pH (5-10), temperature (20-50°C), incubation time (0-60 min), silver precursor concentration (0.5-2 mM), biomass amount (5-10 g) and the ratio of fungal extract to and silver nitrate which determined by elevating the extract amount in 1 mM of metal precursor (1:2). The input factor was represented by two levels: the low (-) and the high (+) level of the range for each parameter. A six variables 16-run screening experimental design of PBD was conducted using Minitab 21 trial software. The level of each parameter was illustrated in Table 1. All trials were performed in duplicate, and the peak intensity average was employed as response of variables. The impact of each variable on the process was determined from the first order reaction:

| Y= β_0_ + ∑ β_i_X_i_ |  |
| --- | --- |

Where Y is the predicted peak intensity, β_i_ and β_0_ are respectively the linear coefficient and the model intercept, X_i_ is the independent variable level.

For the model, a statistical analysis was employed to test the significance of the investigated variables. Factors with the most positive effects were preferred based on Pareto chart results.

**Optimization of the green SNPs synthesis using CCD**

A four variable-four factorial CCD was conducted for the most significant variables. A second order equation mathematical equation was employed to explore the relation between the variation in the peak intensity and the selected variables:

| Y= β_0_ + ∑β_i_X_i_ + ∑β_ii_X_i_ + ∑ β_i_X_j_ |  |
| --- | --- |

Where Y represents the response of peak intensity, β_0,_ β_i_ and β_ii_ are respectively the regression coefficient, the linear model intercept and the coefficient calculated via the model, X_i_ is the coded variable of independent variable. A total of twenty-five runs were generated and allocated in one block with the confidence interval of 95%. Analysis of variance was applied for the model to determine the significance of each examined factor. Surface plots of 3-dimentional response were depicted to clarify the main response and interactive ones between the dependent factors and the independent one.

**Cytotoxicity assay**

Human cells of hepatocellular carcinoma (HepG2), breast cancer (MCF-7) and lung carcinoma (A549) were purchased from ATCC via VACSERA (holding company for biological products and vaccines), Cairo, Egypt.

**References**

Abd El-Ghany, M.N., Hamdi, S.A., Korany, S.M., Elbaz, R.M., Emam, A.N., Farahat, M.G., 2023. Biogenic silver nanoparticles produced by soil rare actinomycetes and their significant effect on *Aspergillus*-derived mycotoxins. Microorganisms 11(4), 1006.

Ahluwalia, V., Kumar, J., Sisodia, R., Shakil, N.A., Walia, S., 2014. Green synthesis of silver nanoparticles by *Trichoderma* *harzianum* and their bio-efficacy evaluation against *Staphylococcus aureus* and *Klebsiella pneumonia*. Industrial Crops and Products 55, 202-206.

Al-Soub, A., Khleifat, K., Al-Tarawneh, A., Al-Limoun, M., Alfarrayeh, I., Al Sarayreh, A., Al Qaisi, Y., Qaralleh, H., Alqaraleh, M., Albashaireh, A., 2022. Silver nanoparticles biosynthesis using an airborne fungal isolate, *Aspergillus flavus*: optimization, characterization and antibacterial activity. Iranian Journal of Microbiology 14(4), 518.

Bhainsa, K.C., D'souza, S., 2006. Extracellular biosynthesis of silver nanoparticles using the fungus *Aspergillus fumigatus*. Colloids and surfaces B: Biointerfaces 47(2), 160-164.

Birla, S.S., Gaikwad, S.C., Gade, A.K., Rai, M.K., 2013. Rapid synthesis of silver nanoparticles from *Fusarium oxysporum* by optimizing physicocultural conditions. The Scientific World Journal 2013.

Chatterjee, K., Taneja, J., Khullar, S., Pandey, A.K., 2023. Antifungal activity of silver nanoparticles on fungal isolates from patients of suspected mucormycosis. Intern Microbiol 26(1), 143-147.

Rose, G.K., Soni, R., Rishi, P., Soni, S.K., 2019. Optimization of the biological synthesis of silver nanoparticles using *Penicillium oxalicum* GRS-1 and their antimicrobial effects against common food-borne pathogens. Green Processing and Synthesis 8(1), 144-156.

Sharaf, E.M., Hassan, A., Al-Salmi, F.A., Albalwe, F.M., Albalawi, H.M.R., Darwish, D.B., Fayad, E., 2022. Synergistic antibacterial activity of compact silver/magnetite core-shell nanoparticles core shell against Gram-negative foodborne pathogens. Frontiers in Microbiology 13.

Sundaravadivelan, C., Padmanabhan, M.N., 2014. Effect of mycosynthesized silver nanoparticles from filtrate of *Trichoderma harzianum* against larvae and pupa of dengue vector Aedes aegypti L. Environmental Science and Pollution Research 21, 4624-4633.

Syed, B., Prasad, N., Dhananjaya, B., Yallappa, S., Satish, S., 2016. Synthesis of silver nanoparticles by endosymbiont *Pseudomonas fluorescens* CA 417 and their bactericidal activity. Enzyme and Microbial Technology 95, 128-136.
